# Role of Architecture in the Function and Specificity of Two Notch-Regulated Transcriptional Enhancer Modules

**DOI:** 10.1371/journal.pgen.1002796

**Published:** 2012-07-05

**Authors:** Feng Liu, James W. Posakony

**Affiliations:** Cell and Developmental Biology Section, Division of Biological Sciences, University of California San Diego, La Jolla, California, United States of America; Harvard Medical School, Howard Hughes Medical Institute, United States of America

## Abstract

In *Drosophila melanogaster*, *cis*-regulatory modules that are activated by the Notch cell–cell signaling pathway all contain two types of transcription factor binding sites: those for the pathway's transducing factor Suppressor of Hairless [Su(H)] and those for one or more tissue- or cell type–specific factors called “local activators.” The use of different “Su(H) plus local activator” motif combinations, or codes, is critical to ensure that only the correct subset of the broadly utilized Notch pathway's target genes are activated in each developmental context. However, much less is known about the role of enhancer “architecture”—the number, order, spacing, and orientation of its component transcription factor binding motifs—in determining the module's specificity. Here we investigate the relationship between architecture and function for two Notch-regulated enhancers with spatially distinct activities, each of which includes five high-affinity Su(H) sites. We find that the first, which is active specifically in the socket cells of external sensory organs, is largely resistant to perturbations of its architecture. By contrast, the second enhancer, active in the “non-SOP” cells of the proneural clusters from which neural precursors arise, is sensitive to even simple rearrangements of its transcription factor binding sites, responding with both loss of normal specificity and striking ectopic activity. Thus, diverse cryptic specificities can be inherent in an enhancer's particular combination of transcription factor binding motifs. We propose that for certain types of enhancer, architecture plays an essential role in determining specificity, not only by permitting factor–factor synergies necessary to generate the desired activity, but also by preventing other activator synergies that would otherwise lead to unwanted specificities.

## Author Summary

Enhancers, or *cis*-regulatory modules, are the major transcriptional control elements in the genome. Some enhancers are activated by the Notch cell–cell signaling pathway, in many different cellular contexts in the same organism. It is well established that the cell type–specific activity of each such enhancer depends in part on its particular combination of transcription factor binding sites. But how important is the enhancer's “architecture”—the number, order, spacing, and orientation of these binding sites—in determining its function and specificity? Here we have examined in detail the role of architecture in the activity of two different Notch-regulated enhancers. We find that one is largely insensitive to rearrangement of its important factor binding sites. But even simple alterations can cause the second enhancer to lose much of its normal activity and to become active in novel territories. By more substantial rearrangements, we can even convert the specificity of the second enhancer to that of the first. Therefore, besides binding site composition, the architecture of some enhancers also plays an essential role in determining when and where they respond to the same signaling pathway during development.

## Introduction

Each of the major developmental signaling pathways is required to effect a large number of conditional cell fate specification events during the development of a single animal species. That the cell fate outcome in each instance is generally distinct presents an enormous regulatory challenge. The pathway must be capable of great specificity, activating in each context only the appropriate subset of its target gene repertoire. This is achieved through the remarkable integrative capacity of the corresponding transcriptional *cis*-regulatory modules [1], [2].

In addition to binding sites for the signaling pathway's transducing transcription factor, each signal-responsive enhancer also includes binding sites for one or more “local activators” — factors expressed specifically in the cells or tissues in which the signaling event will take place [1]. Signaling-dependent activation of the enhancer requires the synergistic action of the transducing factor and the appropriate local activator(s), thus ensuring that only modules with the proper combination of binding sites will function in each context. An enhancer's particular motif combination can thus be thought of as a “code” that helps define the module's specificity.

But while describing a signal-activated enhancer in terms of its characteristic “code” is useful conceptually, it sidesteps a fundamental question. Is the module's activity and specificity determined primarily or solely by the code (in which case a wide variety of binding site arrangements may yield the same output), or instead does the enhancer's particular architecture (the number, order, spacing, and orientation of the motifs) play a critical role [3]?

A number of recent studies have addressed this issue by analyzing the architecture of orthologous *cis*-regulatory modules in different species. It has been found that a remarkable degree of architectural rearrangement is compatible with retention of an enhancer's specificity, but that there is nevertheless a strong tendency for particular combinations of adjacent motifs (“grammar” elements) to be preserved [4]–[8]. This implies that the module's “micro-architectural” features play an essential role in generating its output. A complementary approach is to extensively mutagenize and rearrange a chosen enhancer in an individual species, to determine the effect of specific experimental interventions on its function and specificity. This strategy, when applied to the *sparkling* (*spa*) eye enhancer in *Drosophila melanogaster*, for example, has yielded strong evidence that short-range factor-factor interactions can be critical determinants of a module's specificity [9].

Here we have investigated in detail how two signal-regulated enhancers in *Drosophila* respond to a variety of perturbations to their architecture. Both of these enhancer modules are activated by signaling via the Notch receptor, and both include five high-affinity binding sites for the pathway's transducing transcription factor, Suppressor of Hairless [Su(H)]. Their outputs, however, are entirely distinct. The Autoregulatory Socket Enhancer 5 (ASE5) is active specifically in the socket cell of external sensory organs [10]. It lies just downstream of the *Su(H)* gene itself, and we have found that it is responsible for the long-term maintenance of *Su(H)* autoregulation in socket cells (Liu & Posakony, unpublished) [10]. The “mα enhancer” is located just upstream of the *E(spl)mα* gene (which encodes a member of the Bearded family of proteins) and directs its expression specifically in the “non-SOP” cells of the proneural clusters (PNCs) from which sensory organ precursor (SOP) cells arise; the module is also active in the wing margin zone of the wing imaginal disc [11].

The distinct specificities of the two enhancers are attributable in part to the fact that the mα enhancer, but not ASE5, includes one high-affinity “E box” binding site for Achaete/Scute-class basic helix-loop-helix (bHLH) proneural activator proteins. This motif is required for the module's activity in PNCs [11]. However, we have found that both enhancers include multiple strong binding sites for POU-homeodomain (POU-HD) transcription factors, and that these are essential for the normal activity of each module in its respective domain. This immediately poses the question of how the combinatorial action of Su(H) plus POU-HD factors generates such very different output specificities as socket cells versus wing margin.

Our analysis shows that while the function and specificity of ASE5 is resistant to many different alterations of its architecture, the mα enhancer is highly sensitive. We find that simply exchanging the positions of the E box motif and one of the POU-HD sites profoundly alters the module's specificity. The proneural cluster activity is severely reduced, at the same time that striking ectopic “stripe” specificities in the wing disc are generated. Significantly, weak ectopic activity in socket cells is also now observed. We further find that when the essential transcription factor binding sites of the native mα enhancer are placed in much closer proximity, the module's normal specificity is almost entirely lost, and instead it behaves like ASE5 in displaying strong activity in socket cells. Thus, the specificity of one Notch-responsive enhancer can be converted to that of another by alterations in its organization.

The study presented here demonstrates clearly that the potential for multiple expression specificities that are both unrelated and unwanted can be inherent in an enhancer's particular combination of transcription factor binding motifs. Our findings are most consistent with a model in which the relative positions and spacings of transcription factor binding sites in an enhancer are organized so as to promote functional synergies between activators that generate the desired specificity, while at the same time preventing different activator synergies that would otherwise create undesirable specificities. We discuss the possible implications of these results for our understanding of *cis*-regulatory evolution.

## Results

### Overview of two Notch-regulated enhancer modules with distinct specificities

**Figure 1:**
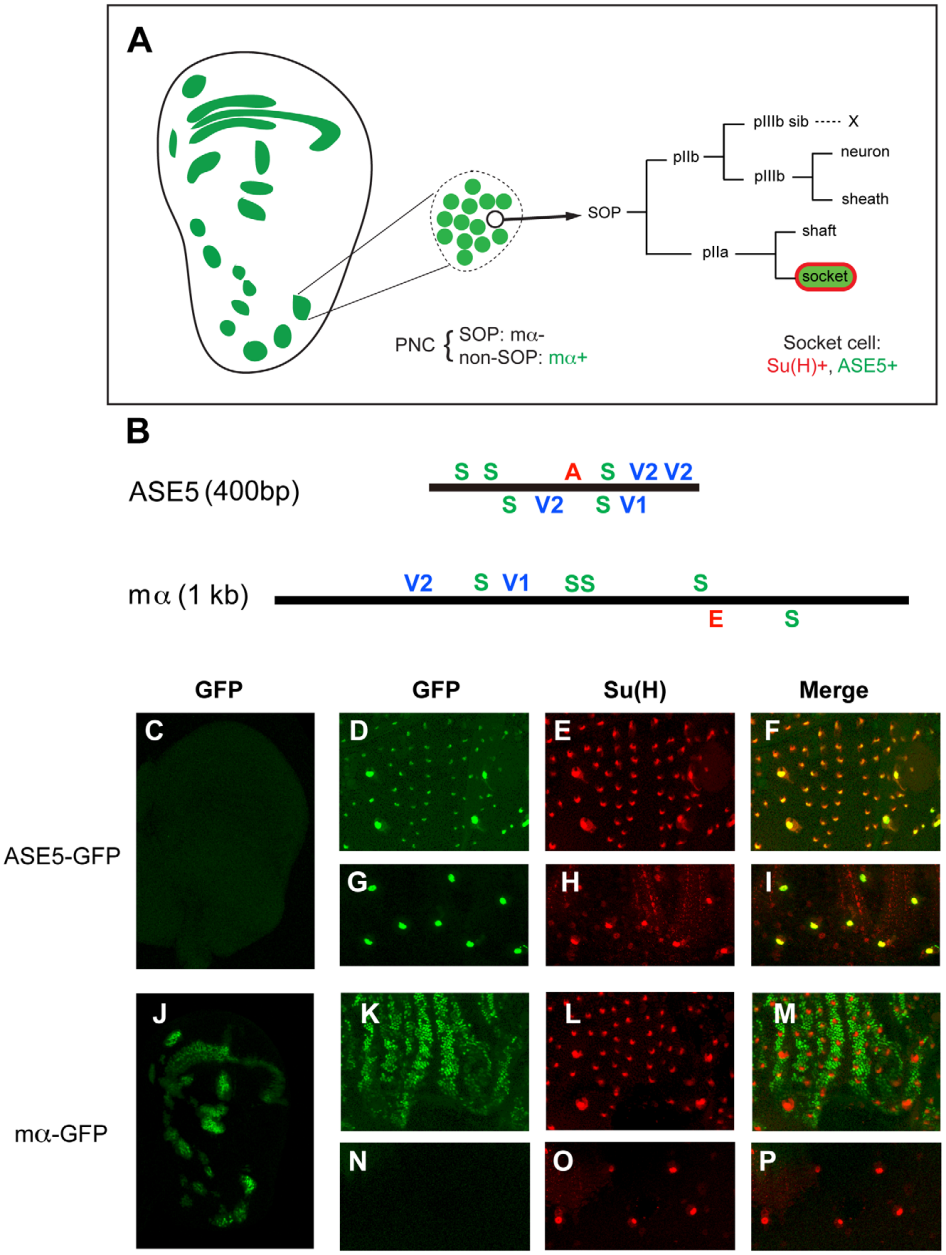
ASE5 and the mα enhancer are active in distinct cell types in development. (A) Diagram showing the relationship between the expression specificities of the mα enhancer and ASE5. Drawing at left represents a late third-instar wing imaginal disc; expression territories of the mα enhancer are shown in green. This enhancer is active primarily in proneural clusters (PNCs), each of which gives rise to a sensory organ precursor (SOP) for one of the external sensory organs of the adult fly. One PNC is shown in expanded form in the middle of the panel, to illustrate that the mα enhancer is active specifically in the “non-SOP” cells of each cluster (green), and not in the SOP (white circle) [11]. The right part of the panel illustrates the cell lineage by which the SOP generates the four cells that make up an external mechanosensory organ. ASE5 is active specifically in one of these post-mitotic progeny cells, the socket cell (green), which is also marked by high-level expression of Su(H) (red) [10]. (B) Diagrams illustrating the architecture of the two transcriptional enhancer modules analyzed in this study. ASE5 is defined by a 0.4-kb genomic DNA fragment [10] (see Text S1), while the mα enhancer is contained within a 1.0-kb fragment [11]. Known transcription factor binding sites within each module are shown. Essential motifs within ASE5 include five high-affinity Su(H) sites (green S), four strong Vvl sites (blue V1, V2), and a single 11-bp sequence (AACGCGAAGCT) designated the A motif (red A). Functional motifs within the mα enhancer include five high-affinity Su(H) sites, two strong Vvl sites, and a proneural protein “E box” site (red E). Motifs are defined as follows: S, YGTGDGAA (TGTGTGAA omitted); V1, RYRYAAAT; V2, AATTAA; E, RCAGSTG. (C–P) Distinct specificities of ASE5 and the mα enhancer are demonstrated by the patterns of GFP reporter expression (green) they drive in transgenic flies at three different developmental stages. Shown are wing imaginal discs of late third-instar larvae (C, J), pupal nota at 24 hours APF (D–F, K–M), and dorsal epithelium of adult abdomen (G–I, N–P). Socket cells of external sensory organs are marked by anti-Su(H) antibody stain (red). Note that *ASE5-GFP* is active specifically in both pupal (D–F) and adult (G–I) socket cells [as marked by Su(H) immunoreactivity], but is inactive in the PNCs of both the third-instar wing disc (compare C to J) and the pupal notum (compare D to K). By contrast, *mα-GFP* is specifically active in PNCs at both stages (J, K) and also exhibits expression in the wing margin territory (J), but is inactive in both pupal — note lack of overlap between green (*mα-GFP*) and red [Su(H)] signals in M — and adult socket cells (N–P).

The two *Drosophila melanogaster* enhancer modules investigated in this study have several features in common (Figure 1A–1B). Each includes five high-affinity binding sites for Su(H), and each is activated in specific cells in response to signaling through the Notch receptor [10], [11]. Each also requires inputs from other transcription factors for its normal activity and specificity (this study) [10], [11].

The 0.4-kb ASE5, which is active specifically in the socket cells of external sensory organs (Figure 1A, 1C–1I), is responsible for the long-term maintenance of *Su(H)* autoregulation in these cells (Liu & Posakony, unpublished) [10]. Using a combination of scanning mutagenesis, yeast one-hybrid screens, and electrophoretic mobility shift assays (EMSAs), we have found that ASE5's activity is dependent on two other types of sequence motif besides the Su(H) sites (see Figures S1, S2, S3, S4, and Text S1). The first is an 11-bp sequence (AACGCGAAGCT) called the A motif, which is located between the third and fourth Su(H) sites (Figure 1B). The other required input comes via multiple conserved binding sites for the POU-HD factor Ventral veins lacking (Vvl), three of which are clustered toward the 3′ end of the enhancer, with a fourth located between the third Su(H) site and the A motif (Figure 1B; Figure S6). We find that the Vvl binding motifs in ASE5 are of two types. The first, represented by the single site designated V1 in Figure 1B (GCATAAAT), resembles previously described Vvl octamer sites [12]–[14] in conforming to the definition RYRYAAAT. The second type, represented by the three sites designated V2, is defined by the hexamer AATTAA. We suggest that these distinct motif classes mediate Vvl binding via the combined POU_S_+POU_H_ domains and by the POU_H_ domain alone, respectively [15].

The 1.0-kb mα enhancer, by contrast, is active in the Notch-inhibited (“non-SOP”) cells of the proneural clusters (PNCs) that give rise to sensory organ precursors (SOPs) of the adult peripheral nervous system (Figure 1A, 1J–1P) [11]. It also drives expression in the wing margin territory that represents the boundary between the dorsal and ventral tissue primordia of the wing (Figure 1A, 1J) [11]. The PNC activity of the enhancer depends critically on direct input from the Ac/Sc proneural transcription factors, mediated by a single high-affinity “E-box” binding site, designated E in Figure 1B [11].

The description thus far is consistent with the hypothesis that the distinct expression specificities of ASE5 and the mα PNC enhancer are attributable to their use of distinct regulatory “codes” (combinations of transcription factor binding motifs): S+A+V in the case of ASE5 and S+E in the case of mα. Under this model, both modules are directly activated by Su(H) in response to Notch signaling, but in different cell types due to their use of different “local activators” [1].

However, this simple interpretation is directly challenged by our recent recognition that the mα PNC enhancer fragment includes conserved Vvl (POU-HD) binding sites of both the V1 and V2 types (Figure 1B; Figure S7). This finding immediately raises the question of why the mα module is not active in socket cells, especially since the S+V motif combination alone is capable of supporting expression in adult socket cells (Figure S4). We will address this problem in the context of our investigation of the role of architecture in the function and specificity of the two enhancers.

### Rearrangement and altered spacing of required motifs has little effect on the activity of ASE5

**Figure 2:**
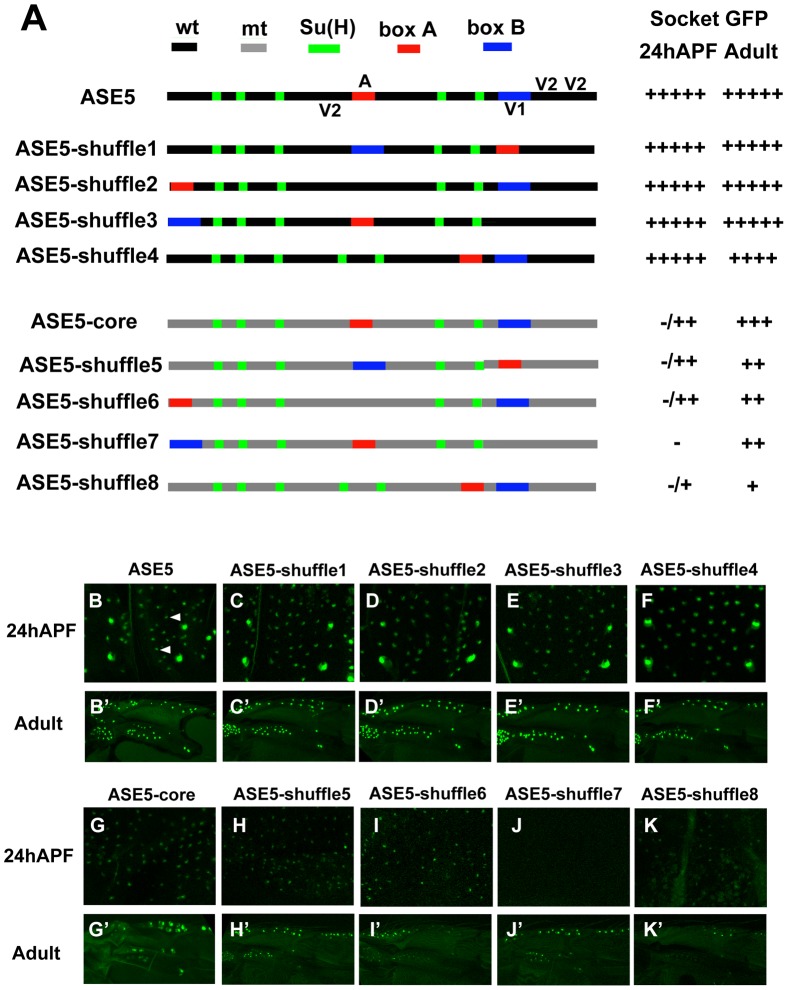
Rearrangement of required sequence elements has little effect on the activity of ASE5. (A) Diagrams of ASE5-GFP reporter gene constructs in the “shuffle” series. The five Su(H) binding sites are marked in green; box A (see text) is in red; box B is in blue. Other wild-type (wt) sequences are shown in black, while mutant (mt) sequence (see Materials and Methods) is marked in gray. All constructs are of the same size as wild-type ASE5; the positions of the box A and box B elements are exchanged with those of similar-sized segments elsewhere in the module. ASE5-shuffle1–4 retain wild-type sequences of ASE5, while ASE5-shuffle5–8 bear mutated sequences between the Su(H) sites, box A, and box B. Observed levels of GFP expression in socket cells are summarized at right. Wild-type ASE5 is scored as very strong (+++++); other constructs vary from very strong to moderate (+++) to very weak (+). Constructs that fail to drive detectable GFP expression are indicated as negative (−). (B–K, B′–K′) Effects of motif rearrangements on the activity of ASE5 are examined in nascent socket cells of notum microchaetes at 24 hours APF (B–K; see arrowheads in B), and in mature socket cells in the anterior proximal wing in adults (B′–K′); results are summarized in (A).

We used a series of enhancer-reporter transgene constructs to investigate whether ASE5's function in the socket cell is dependent on a particular configuration of its essential sequences (Figure 2). These constructs focused on the positions of two small sequence segments (box A and box B), which are centered on the single 11-bp A motif (described above) and on the high-affinity Vvl binding site V1, respectively (Figure 2A). We first generated four variants of ASE5, referred to as ASE5-shuffle1–4, in which the positions of box A, box B, or both are altered (Figure 2A). The rearrangements were designed to alter the locations and distances of box A, box B, and the five Su(H) sites relative to each other. As shown in Figure 2, all four variants exhibit essentially the same functional capacity as the wild-type ASE5; that is, they all drive strong GFP expression specifically in both nascent (Figure 2B–2F) and mature (Figure 2B′–2F′) socket cells in pupal-stage and adult flies, indicating that a particular arrangement of these critical motifs is not required for ASE5's activity or specificity.

To investigate more rigorously the role of architecture in ASE5 function, we tested the effect of making the same sequence rearrangements within an extensively mutated version of ASE5 that retains only box A, box B, and the five Su(H) sites in wild-type form (ASE5-core; Figure 2A). We wondered, for example, whether the presence of additional Vvl binding sites besides V1 might mask the effects of the rearrangements in the context of an otherwise wild-type enhancer. Due to its lack of all but one Vvl binding motif, ASE5-core is only weakly active in nascent socket cells, and moderately active in adult socket cells (Figure 2G, 2G′). We find that all four rearranged versions of ASE5-core (ASE5-shuffle5–8; Figure 2A) are active in adult socket cells (Figure 2H′–2K′). In nascent socket cells, ASE5-shuffle7 is inactive, while the other three versions (ASE5-shuffle-5, -6, and -8) remain weakly active at levels that are comparable to ASE5-core (compare Figure 2G–2K). Thus, even in the context of a significantly weakened version of the enhancer, multiple rearrangements of the critical box A and box B segments are compatible with continued function and specificity of ASE5.

**Figure 3:**
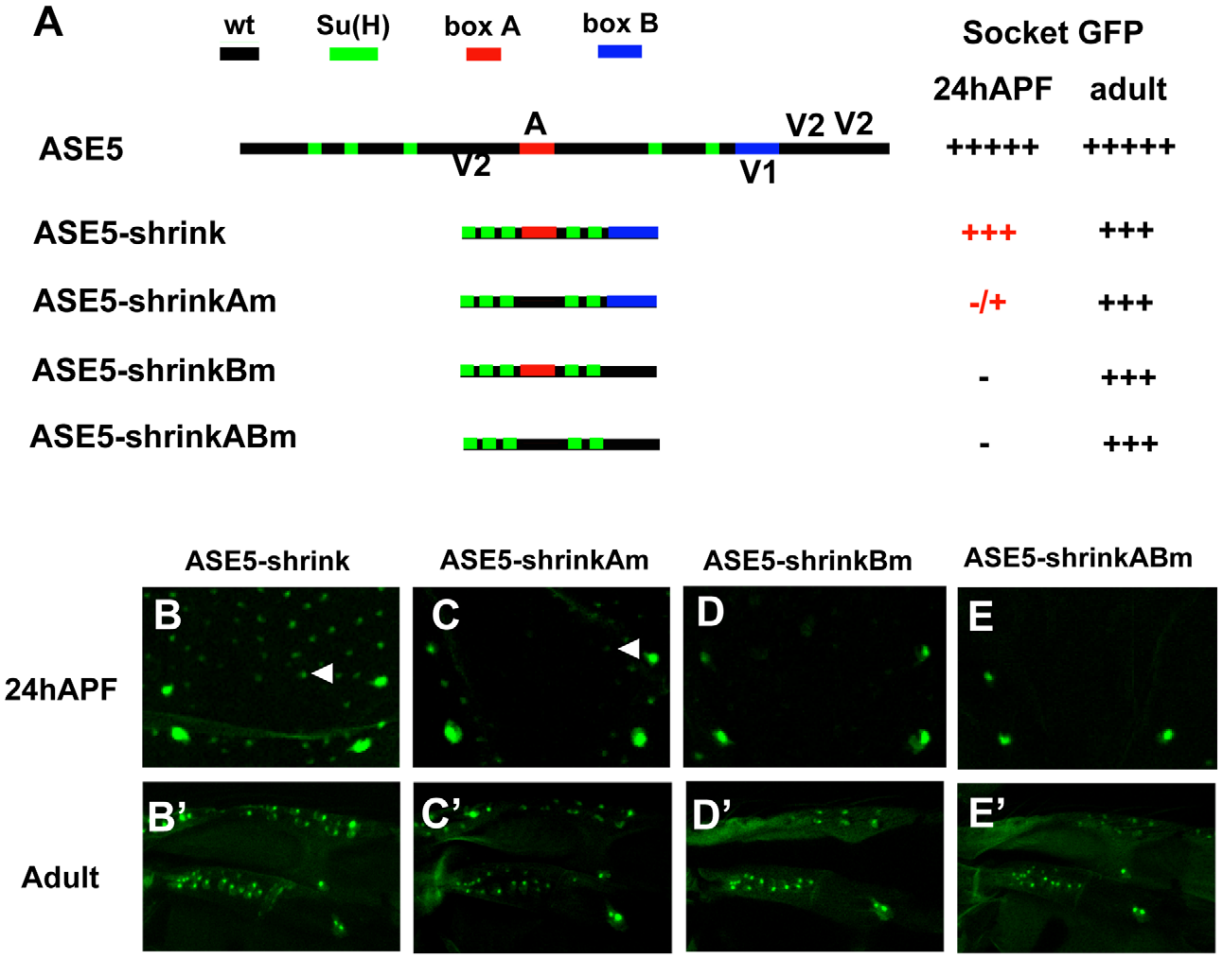
More compact positioning of required motifs in ASE5 increases their activity. (A) Diagrams of ASE5-GFP reporter gene constructs in the “shrink” series; wild-type ASE5 is shown for comparison. The five Su(H) binding sites are marked in green; box A is in red; box B is in blue. Observed levels of GFP expression in socket cells are summarized at right, using the same semi-quantitative scoring system as in Figure 2. (B–E, B′–E′) Reporter gene activities are examined in nascent socket cells of notum microchaetes at 24 hours APF (B–E; see arrowheads in B and C), and in mature socket cells in the anterior proximal wing in adults (B′–E′); results are summarized in (A). By comparison to ASE5-core (see Figure 2), ASE5-shrink (B, B′) is significantly more active in nascent pupal-stage socket cells. Nevertheless, the overall combinatorial logic of the enhancer is retained at this stage, as shown by its dependence on box A (Am; C, C′) and box B (Bm; D, D′). Bringing just the five Su(H) sites close together permits moderate activity in adult but not pupal-stage socket cells (ABm; E, E′), in contrast to the behavior of ASE5M2, which is inactive at both stages (see Figure S1).

The ASE5-shuffle constructs shown in Figure 2 were designed to retain the overall dimensions of the enhancer while rearranging the required motifs. Using sequence-deleted versions of ASE5, we also examined the effect of more global changes in spacing and/or helical phasing between these motifs (Figure 3). To create the ASE5-shrink construct, we deleted all sequences within the enhancer except the five Su(H) sites, box A, box B, and 5 bp flanking each element (Figure 3A). We find that this version of ASE5 largely retains the overall activity and specificity of the wild-type module: It drives robust GFP expression in both nascent and adult socket cells, though at somewhat reduced levels (Figure 3B, 3B′). Moreover, while box A and box B are not required for the activity of ASE5-shrink in adult socket cells (Figure 3C′–3E′), they continue to be essential for its function in nascent socket cells (Figure 3C–3E), indicating that the enhancer's combinatorial logic persists to a large degree even in this greatly compacted version.

Overall, then, the results presented in Figure 2 and Figure 3 indicate that this particular Notch-responsive enhancer module does not rely heavily on a specific architecture for its normal activity.

### More compact positioning of required motifs can substantially augment their activity

The results presented in Figure 3 offer another important insight into the effects of compacting the required motifs in the ASE5 enhancer. First, compared to ASE5-core (Figure 2A, 2G, 2G′), ASE5-shrink is significantly more active in nascent pupal-stage socket cells (Figure 3A, 3B, 3B′). Thus, it appears that positioning the required motifs closer together permits greater synergy between the various inputs, thus elevating the module's output in the early phase of its activity. Likewise, it is noteworthy that the “ABm” version of ASE5-shrink (Figure 3A), which retains only the five Su(H) sites in wild-type form, is robustly active in adult socket cells (Figure 3E′), while ASE5M2, the corresponding version of ASE-core, is completely inactive (Figure S1A, S1D′). Again, reducing the spacing between transcription factor binding sites permits functional synergies that are not observed when the same motifs are at their native distances. Thus, even in a module such as ASE5 that is overall relatively insensitive to alterations of its architecture, the normal spacing of required motifs may act to restrain activities that would otherwise emerge.

### Conserved POU-HD binding sites in the the mα enhancer are required for its normal activity in the wing imaginal disc

**Figure 4:**
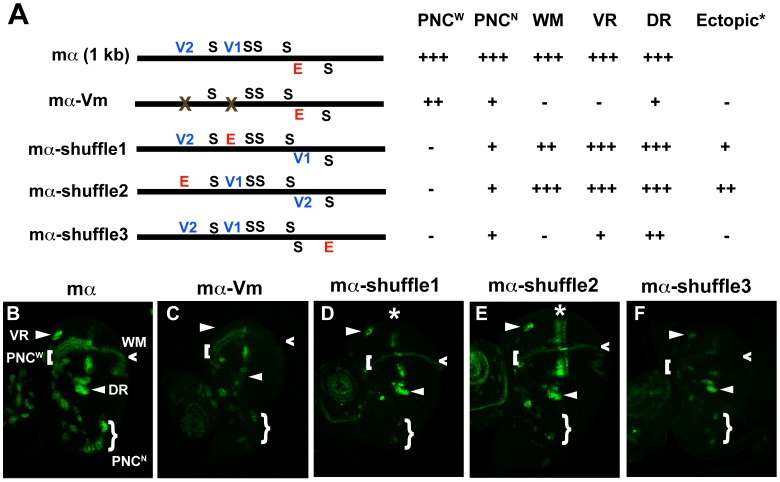
Rearranging required transcription factor binding motifs in the mα enhancer strongly affects its activity. (A) Diagrams of the wild-type mα enhancer and variants. The module's five Su(H) sites (S) are shown in black; the lone “E box” proneural protein binding site (E) is in red; the two Vvl (POU-HD/homeodomain) sites (V1, V2) are in blue. mα-Vm has both Vvl sites mutated, while the mα-shuffle constructs respectively exchange the position of the E motif with those of the V1, V2, and S_1_ sites [11]. Observed patterns and levels of GFP expression driven by each variant are summarized at right. Symbols are as follows (see B): PNC^W^, proneural clusters flanking the anterior wing margin primordium; PNC^N^, proneural clusters of the notum region; WM, wing margin primordium; VR, ventral radius proneural cluster; DR, dorsal radius proneural cluster. (B–F) GFP reporter expression in wing imaginal discs of late third-instar larvae. Among other effects, mutating the Vvl motifs in the enhancer (mα-Vm) eliminates or severely reduces its activity in VR, WM, DR, and PNC^N^ (B, C). Exchanging the position of the E motif with that of either the V1 or V2 site (mα-shuffle1 and mα-shuffle2) severely reduces activity in many PNCs, while also yielding ectopic activity in a stripe within the wing pouch region (asterisk) (B, D–E). Exchanging the positions of the E and S_1_ sites (mα-shuffle3) essentially eliminates PNC^W^ and WM activity, while greatly reducing activity in other proneural clusters (B, F).

In addition to its five high-affinity Su(H) binding sites and single proneural protein binding site, the functional roles of which have been defined previously [11], we observed that the mα enhancer includes two POU-HD binding sites, one each of the V1 octamer (RYRYAAAT) and V2 hexamer (AATTAA) types (Figure 4A). Conservation of these sites in other *Drosophila* species (Figure S7) suggested that they might contribute an important input to the enhancer's activity. Indeed, we find that mutating these two motifs (mα-Vm; Figure 4A) greatly affects the pattern and level of GFP reporter expression driven by the enhancer in the wing imaginal disc (Figure 4B–4C). Expression in most PNCs is substantially reduced, and in certain instances abolished, while activity in the wing margin is eliminated (Figure 4C). Thus, one or more POU-HD and possibly homeodomain factors appear to provide essential activating inputs into the mα enhancer. We note, for example, that although Vvl is expressed strongly in the wing pouch region of the disc, it is not expressed in the wing margin territory [16]. Here, another POU-HD factor, Nubbin, may act through the sites we have identified [17].

### Rearrangement of transcription factor binding sites profoundly affects the spatial specificity of the mα enhancer

We next sought to investigate, as we had for ASE5, the relationship between the architecture of the mα enhancer and its activity and spatial specificity. Again, we tested the functional consequences of rearranging its essential transcription factor binding motifs — five Su(H) sites, one proneural site, and two POU-HD sites (Figure 4A). We constructed three variants of the enhancer, called mα-shuffle1–3, in which the position of the proneural E-box motif is exchanged with that of the V1 site, the V2 site, or the fifth Su(H) site, respectively (Figure 4A). In each case, we observed major alterations in the module's spatial and quantitative patterns of activity in the wing imaginal disc (Figure 4B, 4D–4F; summarized in 4A). Both mα-shuffle1 and mα-shuffle2 exhibit severe reduction, and in some cases elimination, of the wild-type reporter's characteristic pattern of robust expression in various PNCs, with activity in certain clusters remaining at normal or near-normal levels (Figure 4B, 4D–4E). Note, for example, the loss of expression in the chemosensory organ clusters flanking the anterior wing margin, but the retention of expression in the dorsal and ventral radius clusters. In addition, both of these variant constructs exhibit ectopic expression in stripes that lie near the anterior-posterior boundary of the wing pouch; this is especially strong in the case of mα-shuffle2 (Figure 4D–4E). mα-shuffle3 likewise displays a strikingly altered pattern of activity (Figure 4B, 4F). Reporter expression is strongly reduced or eliminated in most or all PNCs, while the wing margin expression is essentially eliminated.

In contrast to ASE5, then, the mα enhancer is highly sensitive to alterations of its native architecture. Even modest rearrangement of its key transcription factor binding sites can very significantly alter its spatial pattern of activity.

### Ectopic activity of rearranged mα enhancers in the socket cell

We return now to the question of why, in the presence of both high-affinity Su(H) and Vvl binding sites that very much resemble those of ASE5, the wild-type mα enhancer is not active in socket cells.

**Figure 5:**
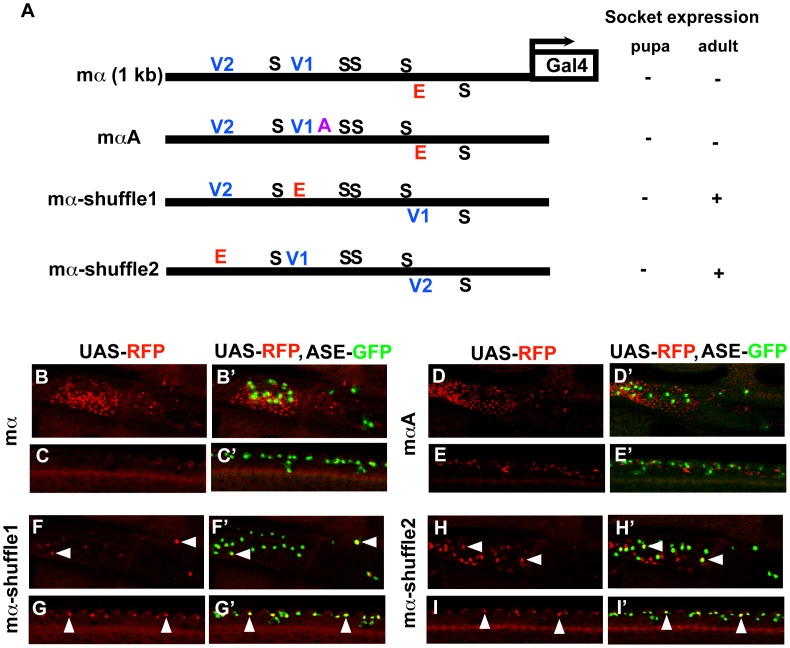
Motif rearrangement in the mα enhancer yields ectopic activity in adult socket cells. (A) Diagrams of the wild-type mα enhancer and variants. The module's five Su(H) sites (S) are shown in black; the lone “E box” proneural protein binding site (E) is in red; the two Vvl (POU-HD/homeodomain) sites (V1, V2) are in blue; the A motif from ASE5 (AACGCGAAGCT; see Figure 1B) is in purple. Via a three-base mutation, the mαA construct adds the A motif to the otherwise wild-type mα enhancer; mα-shuffle1 and mα-shuffle2 are the same site-exchange variants shown in Figure 4. RFP expression driven by each variant (using the GAL4-UAS system to increase sensitivity) in either nascent (pupa; see Figure 6D–6D′ and Figure S5) or mature (adult) socket cells is summarized at right. (B–I, B′–I′) RFP expression (red) driven by mα enhancer variants in adult wing tissue. Top: Region of the campaniform sensilla cluster at the proximal anterior wing margin. Bottom: External sensory organs at the medial anterior wing margin. In merged images (B′–I′), socket cells are identified by expression of an ASE-GFP reporter gene (green) [10]. The wild-type mα enhancer yields only residual RFP expression in surrounding epidermal cells, but not in socket cells (B–C, B′–C′). Adding the A motif from ASE5 (mαA) fails to confer socket cell activity (D–E, D′–E′), while mα-shuffle1 and mα-shuffle2 both drive reporter expression in some adult socket cells (arrowheads in F–I, F′–I′).

The mα-shuffle1 and mα-shuffle2 constructs both exhibit ectopic activity in the wing imaginal disc that may plausibly be interpreted as reflecting strengthened inputs via the V1 and/or V2 motifs (Figure 4D–4E). Accordingly, we asked whether these variant enhancers might be active in at least adult socket cells. Because both mα-shuffle1 and mα-shuffle2 show weak overall activity, we placed each in a GAL4-UAS-based reporter construct to amplify its output (Figure 5). The wild-type mα enhancer never exhibits strong activity in the adult socket cell, even in the GAL4-UAS context (Figure 5B–5C′). Introducing one copy of the A motif from ASE5 — which mediates another important socket cell input — into the otherwise wild-type mα enhancer (mαA; Figure 5A) fails to augment adult socket cell activity (Figure 5D–5E′). By contrast, we readily detected strong, albeit stochastic, expression of both mα-shuffle1 and mα-shuffle2 in socket cells of sensory organs in the adult wing (Figure 5F–5I′). As in the late third-instar wing imaginal disc (see Figure 4), we observe that this gain of activity by mα-shuffle1 and mα-shuffle2 is accompanied by loss of activity in the non-SOP cells surrounding the sensory organs, where the mα enhancer normally functions (compare Figure 5B–5E with Figure 5F–5I). Thus, simply exchanging the positions of the Vvl sites and the E box motif in the module is sufficient to confer a novel socket-cell activity, at the expense of its native specificity. We conclude that the wild-type architecture of the mα enhancer [e.g., the relative positions of the Vvl and Su(H) sites] plays a critical role in inhibiting its activity in socket cells.

### Condensed spacing of essential transcription factor binding sites converts the cell-type specificity of the mα enhancer

**Figure 6:**
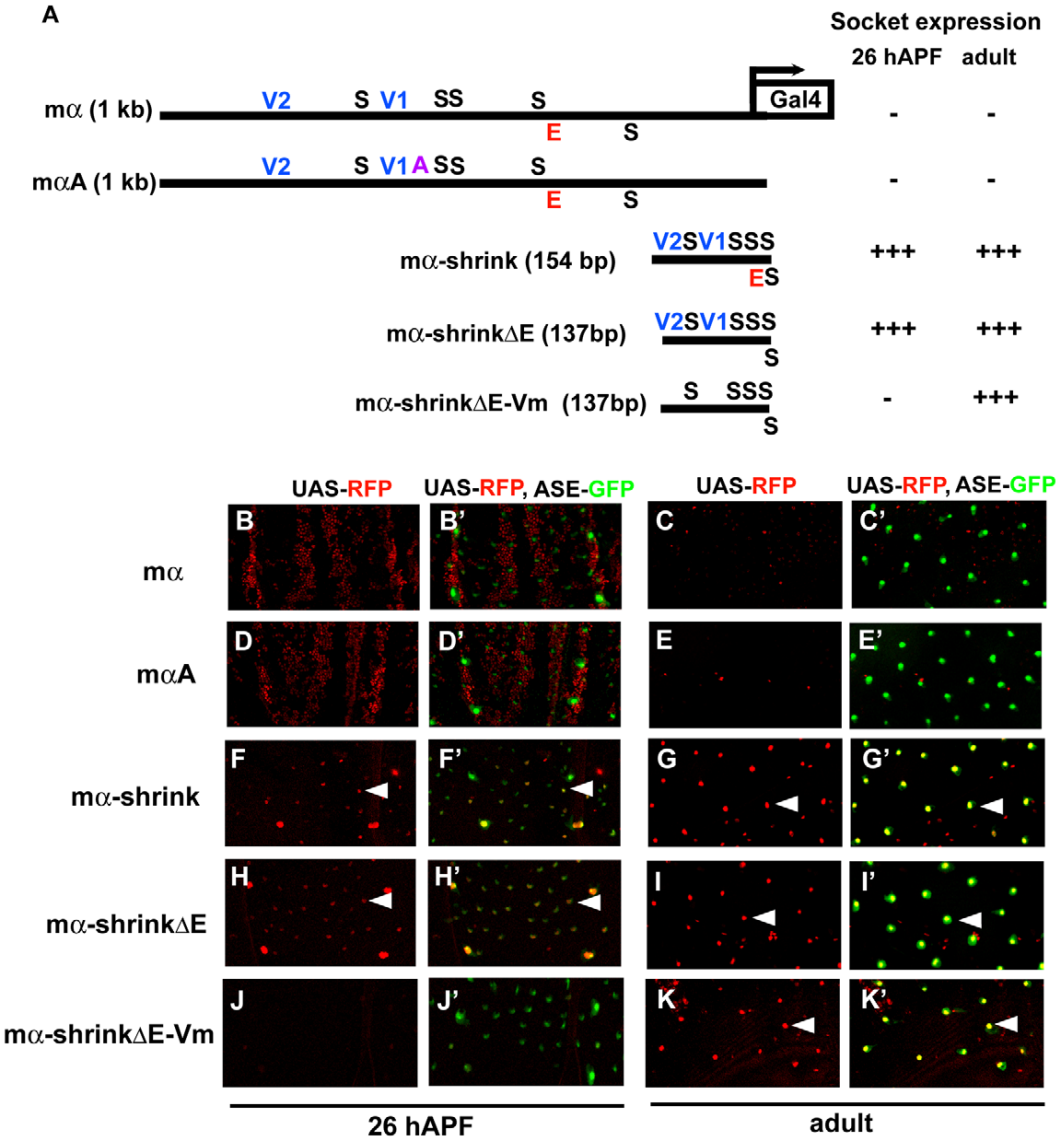
Condensed spacing of transcription factor motifs converts the cell-type specificity of the mα enhancer. (A) Diagrams of the wild-type mα enhancer and variants. The module's five Su(H) sites (S) are in black; the “E box” proneural protein binding site (E) is in red; the two Vvl (POU-HD/homeodomain) sites (V1, V2) are in blue; the A motif from ASE5 (AACGCGAAGCT; see Figure 1B) is in purple. mαA is the same construct shown in Figure 5; the mα-shrink series are synthetic enhancer constructs that include the known essential transcription factor binding sites of the wild-type mα enhancer. RFP expression driven by each construct (using the GAL4-UAS system) in either nascent (pupal-stage; 26 hours APF) or mature (adult) socket cells is summarized at right. (B–K, B′–K′) RFP expression (red) driven by mα enhancer constructs in pupal nota at 26 hours APF (B, B′; D, D′; F, F′; H, H′; J, J′) and dorsal epithelium of adult abdomen (C, C′; E, E′; G, G′; I, I′; K, K′). In merged images (B′–K′), socket cells are identified by expression of an ASE-GFP reporter gene (green) [10]. Arrowheads indicate examples of RFP-expressing socket cells. Both the wild-type mα enhancer (mα) and the mαA variant display residual activity in the non-SOP cells of microchaete proneural clusters of the pupal notum (B, B′; D, D′), but are inactive in both nascent and mature socket cells (B–E, B′–E′). By contrast, the “wild-type” synthetic construct mα-shrink lacks proneural cluster activity (F, F′) and instead drives robust expression in socket cells at both stages (F–G, F′–G′). This activity does not require the E box motif (mα-shrinkΔE; H–I, H′–I′), but mutation of the Vvl sites eliminates activity in nascent socket cells (mα-shrinkΔE-Vm; J, J′). As in the case of the “ABm” version of ASE5-shrink (see Figure 3), the five Su(H) sites of the mα enhancer are sufficient to drive expression in adult socket cells when brought sufficiently close together (K, K′).

To investigate further the role of architecture and motif spacing in determining the specificity of the mα enhancer, we constructed a deletion version of the module (mα-shrink; Figure 6A) by removing all sequences except the five Su(H) sites, the two Vvl sites, the E-box proneural protein binding site, and 5 bp flanking each motif. In this variant, essential transcription factor binding sites in the native enhancer are placed in much closer proximity. Strikingly, while mα-shrink shows very little of the wild-type enhancer's native activity — e.g., it no longer functions in the PNCs for notum microchaetes — it drives robust GFP reporter expression in both nascent and fully differentiated socket cells, closely mimicking the normal activity of a socket cell-specific enhancer, ASE5 (Figure 6B–6C′, 6F–6G′).

To determine which transcription factor binding motifs are largely responsible for mα-shrink's novel specificity, we first deleted the E-box (mα-shrinkΔE; Figure 6A) and found that it makes no significant contribution to the socket cell activity (Figure 6H–6I′). However, adding mutations in the two Vvl motifs to this construct (mα-shrinkΔE-Vm; Figure 6A) leads to nearly complete loss of activity in nascent socket cells, but not in differentiated adult socket cells (Figure 6J–6K′). Recall that the structurally similar ABm version of ASE5-shrink, which also has five Su(H) sites and no Vvl sites, is likewise robustly active in adult, but not pupal-stage, socket cells (see Figure 3). Thus, just as for ASE5 and ASE5-shrink, mα-shrink's novel socket-cell specificity does depend critically, in the early stages of the cell's development, on input via the module's native Vvl sites. These results further demonstrate the essential role of enhancer architecture and motif positioning in generating native specificities and restraining unwanted ones.

## Discussion

### Differential role of architecture in controlling the function and specificity of two Notch-regulated enhancers

Our detailed analysis of two different Notch-regulated transcriptional enhancer modules has revealed that they are very differently dependent on a particular architecture for their activity and specificity. The socket cell-specific ASE5 enhancer tolerates a variety of rearrangements of its required motifs without appreciable alteration of function in either nascent or mature sockets. Even when ASE5 is impaired quantitatively as a result of mutating all of its non-essential sequences, motif rearrangement generally has only modest effects on activity level, and never modifies the enhancer's specificity. In contrast, we found that the mα enhancer is sensitive to simple exchanges in the positions of transcription factor binding motifs, responding with both loss of normal spatial specificity and ectopic activity.

Broadly speaking, then, one might say that ASE5 is more representative of a “billboard” model of enhancer architecture (which posits that transcription factor binding motifs contribute to enhancer function largely independently of how they are organized), while the mα enhancer might be thought of as conforming more closely to an “enhanceosome” model (which suggests that a module's function is crucially dependent on a particular configuration of transcription factor binding sites in order to create synergy between their inputs) [3], [18].

It is useful to consider the characteristics that may determine whether a given module is more likely to lie at the “billboard” or the “enhanceosome” end of the spectrum. Though ASE5 and the mα enhancer are both Notch-activated, they function in different biological contexts, and we suggest that this may be relevant to their respective architectural constraints. ASE5 acts in a single post-mitotic, differentiated cell type to establish and maintain autoregulation of *Su(H)* for several days. Here, due to the availability of cell type-specific “local activators” such as Vvl, and the strong contribution that high Su(H) levels alone can make to the enhancer's activity, the need for a constrained architecture may be quite minimal. The mα enhancer, on the other hand, is faced with the challenging task of rapidly and transiently (over a period of hours) activating expression of the *E(spl)mα* gene in multiple non-SOP cells per PNC, while at the same time repressing its expression in each SOP. This might be expected to create a stringent requirement for constrained spacing between the lone proneural protein binding site and one or more Su(H) sites. At the same time, other aspects of the enhancer's normal specificity rely on inputs via POU-HD and/or homeodomain binding sites — yet these must not be permitted to promote inappropriate activity in socket cells. Again, particular binding motif configurations may be called for as a preventative (see below). The overall point is that two parameters — an enhancer's specific biological task and context, and its particular combination of factor binding sites — are likely to play a major role in determining the architectural constraints to which it may be subject.

### Codes and architectures as determinants of enhancer activity

The case of the mα enhancer serves to underscore the insufficiency, in many instances, of a transcription factor binding site “code” in predicting the specificity of a *cis*-regulatory module [9]. Despite the presence of five Su(H) sites and two motifs that can be bound by Vvl, the native mα enhancer shows no meaningful activity even in adult socket cells. Yet the mα-shuffle1 and mα-shuffle2 variants, in which the positions of the Vvl motifs are altered, do exhibit substantial adult socket cell activity. Thus, it is specifically the wild-type enhancer's architecture that normally prevents this from happening. A similar conclusion derives from examining the functionality of the proneural (E) plus Su(H) (S) “code” embodied in the mα enhancer [11], [19]–[21]. When the lone E box site is in its native and evolutionarily conserved position 14 bp away from one of the Su(H) sites [11], it provides sufficient input to drive robust expression in all wing disc PNCs. But when it is moved instead to the location of one of the Vvl sites, the module's PNC activity is severely reduced. Again, the simple presence of Su(H) and proneural binding motifs in the mα enhancer does not suffice to predict its specificity; rather, the specific arrangement of these sites has a profound effect on its ability to generate the PNC specificity.

### Enhancer architecture/organization serves to restrain inappropriate specificities

The critical role of binding site spacing and organization in generating the transcription factor synergies necessary for the normal activity of many enhancers is becoming increasingly clear. But our mutational analyses of both ASE5 and the mα enhancer demonstrate an equally important role for architecture in preventing inappropriate synergies and hence inappropriate specificities.

Two ASE5 variants are particularly informative in illuminating the importance of motif spacing in restraining enhancer activity. We found that ASE5M2, in which only the five Su(H) sites are intact but spacing is preserved, is completely inactive in both pupal and adult socket cells. By contrast, the ABm version of ASE5-shrink, which likewise retains only the five Su(H) sites but now places them much closer together, is strongly active in adult sockets. Thus, ASE5's native architecture serves in part to prevent the Su(H) sites from responding on their own, and in this way maintains the enhancer's dependence on inputs from the box A and/or box B sequence elements, even in adult socket cells.

Next, the wholly ectopic responsiveness of mα-shrink in both pupal and adult socket cells demonstrates clearly that the potential for unrelated and unwanted specificities can be inherent in an enhancer's particular combination of transcription factor binding motifs. Even as it functions in an inappropriate cell type, mα-shrink follows a recognizable regulatory logic. Its activity in nascent socket cells is fully dependent, as expected from ASE5, on its POU-HD and/or homeodomain sites (and not on its “E box” proneural protein binding site), while its robust adult socket activity — as in the case of the ABm version of ASE5-shrink — requires only the five Su(H) sites.

Finally, the far more modest alterations represented by the “shuffle” versions of the mα enhancer explicitly demonstrate the critical role that motif placement and spacing may have in suppressing inappropriate specificities. Simply exchanging the position of one of the module's “Vvl” sites with that of the E box proneural site creates novel activities in both the wing imaginal disc and the socket cell.

In a recent report, Swanson et al. identified short-range transcriptional repression as the mechanism that prevents the cone cell-specific *sparkling* (*spa*) eye enhancer, which serves the *Drosophila dPax2* gene, from being ectopically active in nearby photoreceptor cells [9]. In this instance, moving the repression-mediating sequences out of their native context apparently eliminated their ability to exert a repressive effect, permitting the module to be active in an inappropriate cell type.

We believe that our results with the mα enhancer are most simply consistent with a different mechanism for restraining unwanted enhancer specificities. In this model, the relative positions and spacings of transcription factor binding sites are organized so as to promote functional synergies between activators that generate the desired specificity, while at the same time preventing different activator synergies that would otherwise create undesirable specificities. Note that, while this mechanism places definite constraints on the allowable motif locations in the module, it does not require that the enhancer be transcriptionally repressed in the incorrect cell type(s).

We cannot strictly rule out the possibility that, despite their simplicity, both of the “site switches” embodied in the mα-shuffle1 and mα-shuffle2 constructs have disrupted the interaction of a short-range repressor with its target activator(s). However, we think this is unlikely for a number of reasons. For example, such a repressor would have to be active in both a broad zone of wing disc tissue and in socket cells — two very different settings. We suggest instead that the most parsimonious explanation for our findings is the synergy promotion/prevention model described above.

What might determine whether a given enhancer makes use of active repression to limit its specificity, or instead utilizes a simpler synergy prevention mechanism? One reasonable possibility is that repression is required, or more common, when the ectopic specificity that must be prevented consists of a cell or cells that are very closely related developmentally to those in which expression is wanted. Such inappropriate cells may be spatially very close to the correct cells, and/or may have a high degree of similarity in their developmental histories and gene expression profiles. In such cases, it may be difficult or impossible to evolve a motif architecture that simultaneously allows the proper activity and prevents the improper. On the other hand, when the ectopic specificity is a very different cell type or tissue, distant both temporally and spatially from the correct one, and sharing very little developmental history, perhaps motif arrangements that act to prevent inappropriate synergies are easier to evolve. Under this rubric, the use of repression by modules as different as the *eve* stripe 2 and *spa* enhancers is readily understood, just as the mα enhancer might instead be expected to inhibit socket cell activity by prevention of the necessary activator synergy. Indeed, the mα module appears to make use of both mechanisms: Activity of this enhancer in the SOP cell is antagonized by repression mediated by Su(H) [11]. As a member of the PNC, the SOP is of course surrounded by, and very closely related to, the non-SOPs.

Finally, it is interesting to consider what characteristics of an enhancer might put it particularly at risk for ectopic activity, which in turn would require the use of the preventive mechanisms we have considered. Certainly utilizing transcription factors that are broadly expressed and active [such as Su(H)] would contribute to such a need, as would using inputs from factors that are members of paralogous families with very similar DNA-binding specificities (e.g., POU-HD proteins).

### Enhancer evolution

The results described here have, we believe, important implications for our understanding of enhancer evolution. It appears that, due to the specific combination of transcription factor binding motifs they employ, some — perhaps most — enhancers harbor the hidden potential to generate certain novel specificities that can be revealed through comparatively simple sequence changes. In a sense, such enhancers are “poised” to express these silent specificities. Depending on how widespread this phenomenon is among enhancers in the whole genome, a tremendous potential may exist to explore a vast “specificity space” through modest mutational events. Moreover, when applied to an individual enhancer, this perspective suggests that a particular novel specificity — one that requires only relatively minor changes in motif placement to be expressed — might be seen to evolve independently in more than one lineage.

Our results also suggest that the minimum size of a given enhancer module may be subject to significant constraints, due to the need to prevent unwanted activator synergies through motif spacing. Thus, even if not all sequences in the enhancer mediate transcription factor inputs, some may be preserved evolutionarily in order to maintain distance between transcription factor binding sites.

## Materials and Methods

### Reporter transgene constructs

Wild-type and mutant ASE5 sequences were synthesized by recursive polymerase chain reaction (PCR) [22]. The two flanking primers contain an EcoRI site and a BamHI site, respectively, which were used to clone the PCR product into the multiple cloning site (MCS) of either the insulated GFP reporter vector pH-Stinger [23] or a modified version of pH-Stinger that carries the attB sequence downstream of the GFP reporter gene (pH-Stinger-attB; gift of Steve Miller, UCSD) (Text S1). ASE5 mutants and other variants, including the “shuffle” and “shrink” series, were generated using oligonucleotides containing specific mutations in the target sequences.

The mα enhancer is contained within a 1.0-kb BamHI-XhoI genomic DNA fragment upstream of the *E(spl)mα* gene [11]. We note that we have examined the activity of two 0.5-kb segments representing the left and right halves of the full 1.0-kb fragment (B. Castro and J.W.P., unpublished). The left sub-fragment includes three Su(H) sites and both POU-HD sites (see Figure 1B), while the right contains two Su(H) sites and the E-box. We find that the left piece fails to drive expression in imaginal disc tissue, and thus does not function as an independent enhancer. The right piece drives reporter expression in PNCs, but at substantially weakened levels, particularly in the notum region of the wing disc. These results strongly suggest that the mα enhancer is best defined as including all five Su(H) sites, and prompted us to carry out our architecture experiments using the full 1.0-kb fragment.

For this study, the wild-type mα enhancer was cloned by standard PCR, with an EcoRI site introduced on the 5′ end and a BamHI site on the 3′ end. The PCR product was digested with these two enzymes and cloned into the MCS of the pH-Stinger-attB vector. The Vvl site-mutant version (mα-Vm) and the “shuffle” variants of the enhancer (mα-shuffle1–3) were generated by overlap extension PCR [24], [25] and cloned into the pH-Stinger-attB vector.

The mα-shuffle1 and mα-shuffle2 variants were also cloned into the vector pH-Gal4 [11]. Three point mutations were introduced into the enhancer by overlap extension PCR to generate mαA, while the “shrink” series of mα enhancer variants were generated by recursive PCR; these constructs were all cloned into pH-Gal4.

Detailed descriptions of the enhancer sequences analyzed in this study are included in Text S1.

### Molecular dissection of ASE5

To map the functionally important sequence motifs in ASE5, we first performed a comprehensive scanning mutagenesis (using non-complementary transversions: A↔C and G↔T) of the sequences between the five Su(H) binding sites, assaying the mutant modules in enhancer-GFP reporter constructs in transgenic flies. We identified four 20- to 30-bp fragments that are essential for ASE5's activity during development. Next, using yeast one-hybrid screens and electrophoretic mobility shift assays (EMSAs), we determined that three of the four fragments contain binding motifs for the POU-HD factor Ventral veins lacking (Vvl). The fourth fragment does not appear to contain a recognizable binding motif for a known transcription factor. By progressive targeted mutagenesis of this fragment, we determined that its activity resides in an 11-bp sequence which we call box A.

We note that, while our analysis has succeeded in identifying what we believe are major inputs responsible for ASE5's activity, it is highly likely that other, unknown factors contribute as well. It should also be noted that our scanning mutagenesis scheme introduced into all ASE5 variants mutated between the second and third Su(H) sites [including ASE5-core and ASE5-shuffle5–8; see Figure 2A; see also Text S1] a new motif (ACAGGTG) fitting our RCAGSTG proneural “E-box” definition. This is evidently of little functional consequence, since (as shown in Figure 2G–2K) these variants are inactive in the PNCs of the pupal notum.

Details of the scanning mutagenesis, yeast one-hybrid screens, and EMSAs are provided in Text S1.

### Fly work

Transgenic enhancer-reporter constructs in the pH-Stinger and pH-Gal4 vectors were co-injected with Δ2–3 helper plasmid into *w^1118^* embryos following the standard protocol [26]. At least five independent insertion lines were obtained and analyzed for each of these constructs. Enhancer-reporter constructs in the pH-Stinger-attB vector were injected into the attP2 line, which carries an attP docking site at 68A4 on the third chromosome and the φC31 integrase gene on the X chromosome [27]. Transgenic flies were identified and homozygosed by eye color (w^+^).

All fly crosses were carried out at 25°C. For timed dissections, white prepupae were picked individually and cultured in a humidified chamber at 25°C to the desired developmental stage.

### Immunostaining and confocal microscopy

For antibody staining, pupal nota and adult abdominal epithelia were dissected in PBT (1× PBS, 0.1% Triton X-100), fixed in 4% paraformaldehyde in PBT for 30 minutes at room temperature, washed, and incubated with rabbit anti-Su(H) antibody (diluted 1∶1000; Santa Cruz Biotechnology, Inc.), followed by anti-rabbit-Alexa555 secondary antibody (diluted 1∶200; Molecular Probes, Invitrogen), and mounted on slides for imaging.

For direct examination of GFP and RFP expression, pupal nota and adult wings were dissected in PBT, fixed in 4% paraformaldehyde in PBT for 30 minutes at room temperature, washed three times, and mounted for imaging.

Images were obtained with a Leica TCS SP2 confocal microscope. Each image presented is an average projection of a series of Z-section images taken at 2-µm intervals.

### Semi-quantitative scoring of reporter gene expression

Levels of GFP or RFP reporter expression (apparent brightness) in socket cells or in specific wing disc territories were scored semi-quantitatively in a range from very strong (+++++) to moderate (+++) to very weak (+). The activities of the wild-type ASE5 and mα enhancers were used as the respective standards for very strong (+++++). Constructs that failed to drive detectable reporter expression were scored as negative (−).

## Supporting Information

Figure S1 Identification of functionally important sequence elements in ASE5. (A) Diagrams of scanning mutagenesis variants of ASE5 tested in GFP reporter gene constructs. The module's five Su(H) binding sites are marked in green; other wild-type (wt) sequences are shown in black, while mutant (mt) sequence (see Materials and Methods) is marked in gray. All constructs are of the same size as wild-type ASE5. Observed levels of GFP expression in socket cells are summarized at right. Wild-type ASE5 is scored as very strong (+++++); other constructs vary from very strong to moderate (+++) to very weak (+). Constructs that fail to drive detectable GFP expression are indicated as negative (−). (B–I, B′–I′) Effects of scanning mutagenesis on the activity of ASE5 are examined in nascent socket cells of notum microchaetes at 24 hours APF (B–I; see arrowhead in B), and in mature socket cells in the anterior proximal wing in adults (B′–I′; see arrowhead in B′); results are summarized in A. Mutating all five Su(H) binding sites (M1) completely abolishes the activity of ASE5 (C, C′). Likewise, mutating all sequences between the Su(H) sites (M2) abolishes ASE5's activity (D, D′). Separately mutating two distinct fragments [fragment X or Y (see A); M10 and M11, respectively] results in severe reduction of ASE5's activity in nascent socket cells, but not in adult socket cells (E–F, E′–F′). Mutating both fragments X and Y (M14) results in complete loss of ASE5-GFP expression at both stages (I, I′).
(TIF)

Figure S2 Combinatorial activation of ASE5 in the developing socket cell. (A) Diagrams of ASE5 mutants containing wild-type sequences of the five Su(H) binding sites (marked in green), along with wild-type (wt) sequences of other specific segments of the enhancer (shown in black); all other sequences are mutant (mt, marked in gray). All variants are of the same size as wild-type ASE5. Enhancer activities of each fragment were tested in GFP reporter gene constructs. Observed levels of GFP expression in socket cells are summarized at right, using the same semi-quantitative scoring system as in Figure S1. (B–I, B′–I′) Reporter gene expression was examined in nascent socket cells of notum microchaetes at 24 hours APF (B–I; see arrowhead in D), and in mature socket cells in the anterior proximal wing in adults (B′–I′; see arrowhead in D′); results are summarized in (A). Inputs from the Su(H) sites plus either fragment X or Y (M18, M20) are sufficient to activate GFP expression in adult socket cells, but are insufficient in nascent socket cells (B–C, B′–C′). Inputs from the Su(H) sites plus both X and Y (M24) are sufficient to activate GFP expression in socket cells at both stages (D, D′). Three sub-elements of Fragment Y (boxes B, C, and D) each contribute to its activity [B: compare M18 (B, B′) and M28 (G, G′); C: compare M25 (see A) and M28 (G, G′); D: compare M20 (C, C′) and M23 (see A)]. Fragment X's function maps to an 11-bp element called the A motif [box A; compare M25 (see A), M29 (H, H′), and M30 (I, I′)]. Fragments X and Y were each used as bait in yeast one-hybrid screens (see Text S1, Table S1, and Table S2).
(TIF)

Figure S3 ASE5 contains multiple Vvl binding sites. (A) Diagram of ASE5, showing positions of strong Vvl binding sites. Su(H) binding sites (S) are marked in green; the A motif is shown in red; boxes B–D are shown in blue. The single type 1 Vvl octamer site, which conforms to the definition RYRYAAAT, is located within box B and is indicated as V1. The type 2 Vvl sites, which all contain the hexamer AATTAA, are indicated as V2; note that one such site is located within both box C and box D. Positions of oligonucleotide sequences used as probes in electrophoretic mobility shift assays (EMSAs) are shown. (B) Competition EMSA using Vvl probe 1 and purified GST-Vvl. A 500-fold excess of unlabeled oligonucleotides were used as competitors (wt, wild-type; mt, mutant). Positions of free and bound probe are indicated. In the probe sequences below, wild-type bases are in upper case; mutant positions are in lower case; the wild-type V1 motif is shown in red. (C) Direct-binding EMSA using wild-type and mutant Vvl probes 1–4 and GST-Vvl. Type 1 Vvl sites are shown in red; type 2 sites are in purple. Mutated bases in mutant probes are indicated with asterisks. Note that probe 2 includes a strong type 2 Vvl binding site and an overlapping weak type 1 site that is a one-base mismatch to the RYRYAAAT motif definition. Both sites are mutated in probe2mt.
(TIF)

Figure S4 Necessity and sufficiency of Vvl binding sites for ASE5's function. (A) Diagram showing wild-type ASE5, three variants (M31–M33) bearing mutated Vvl sites, and two variants (ASE5-SV and ASE5-SV-2) testing the sufficiency of Vvl sites to synergize with Su(H) sites. ASE5M2, which retains in wild-type form only the five Su(H) sites, is also shown (see Figure S1). Su(H) binding sites (S) are marked in green; the position of the A motif (A) is also shown. Type 1 and type 2 Vvl binding sites are indicated as V1 and V2, respectively. Wild-type (wt) segments of the enhancer are shown in black; mutant (mt) segments are marked in gray. All variants are of the same size as wild-type ASE5. Enhancer activities of each variant were tested in GFP reporter gene constructs; observed levels of GFP expression in socket cells are summarized at right, using the same semi-quantitative scoring system as in Figure S1. (B–F, B′–F′) Reporter gene expression was examined in nascent socket cells of notum microchaetes at 24 hours APF (B–F; see arrowheads in C, D), and in mature socket cells in the anterior proximal wing in adults (B′–F′); results are summarized in (A). (B, B′) Mutating the four Vvl motifs shown in A (M31; purple X's) results in loss of ASE5 activity in nascent, but not adult, socket cells. (C–D, C′–D′) Mutating only the type 1 or type 2 Vvl binding sites (M32, M33) greatly weakens, but does not eliminate, ASE5's activity in nascent socket cells; activity in adult socket cells is unaffected. (E–F, E′–F′) Retaining the wild-type sequences of only the five Su(H) sites and either all four (ASE5-SV; E, E′) or just two (ASE5-SV-2; F, F′) Vvl sites is sufficient to drive GFP expression in adult socket cells.
(TIF)

Figure S5 mα-shuffle1 and mα-shuffle2 are not active in nascent socket cells. (A–D) RFP expression (red) driven (using the GAL4-UAS system) by two mα enhancer variants, mα-shuffle1 and mα-shuffle2 (see Figure 5), in the pupal notum at 26 hours APF. In merged images (B, D), socket cells are identified by expression of an ASE-GFP reporter gene (green) [10]. As in the adult (see Figure 5), mα-shuffle2, but not mα-shuffle1, drives limited RFP expression in surrounding epidermal (probably non-SOP) cells. Neither variant drives expression in nascent socket cells.
(TIF)

Figure S6 Alignment of ASE5 enhancer sequences from 12 *Drosophila* species. ASE5 enhancer region sequences were retrieved from the UCSC genome browser (genome.ucsc.edu) and aligned using GenePalette (www.genepalette.org) [28]. Filled boxes represent perfectly conserved words of 9 bases or longer. S: Su(H) binding sites (YGTGDGAA, TGTGTGAA omitted). V1: Type 1 Vvl binding site (RYRYAAAT). V2: Type 2 Vvl binding site (AATTAA). Note the complete conservation of the four Vvl sites (one V1 and three V2) identified in Dmel.
(TIF)

Figure S7 Alignment of mα enhancer sequences from 12 *Drosophila* species. mα enhancer region sequences were retrieved from the UCSC genome browser (genome.ucsc.edu) and aligned using GenePalette (www.genepalette.org) [28]. Filled boxes represent perfectly conserved words of 10 bases or longer. S: Su(H) binding sites (YGTGDGAA, TGTGTGAA omitted). V1: Type 1 Vvl binding site (RYRYAAAT). V2: Type 2 Vvl binding site (AATTAA). As previously described [11], the enhancer's Su(H) sites are conserved, with the exception of the second site from the left (S_4_ [11]) which is lost in Dana, Dwil, Dmoj, Dvir, and Dgri. Note the strict conservation of sequence and spacing in the module's S+E motif combination [11]; this appears to be a critical “grammar” element within the enhancer (this study). Also note that, with the exception of V1 in Dana [which has a single-base mismatch to the definition (ATACAAAC), shown as lower-case “v”], both Vvl binding sites identified in Dmel are fully conserved.
(TIF)

Table S1 Yeast one-hybrid screen using Fragment Y as bait.
(PDF)

Table S2 Yeast one-hybrid screen using Fragment X as bait.
(PDF)

Text S1 Supplemental Methods.
(PDF)
