# Supplementary figures and images for: Role of Architecture in the Function and Specificity of Two Notch-Regulated Transcriptional Enhancer Modules
Source: PLoS Genet. 2012 Jul 5;8(7):e1002796. doi: 10.1371/journal.pgen.1002796 (PMC3390367; doi:10.1371/journal.pgen.1002796)

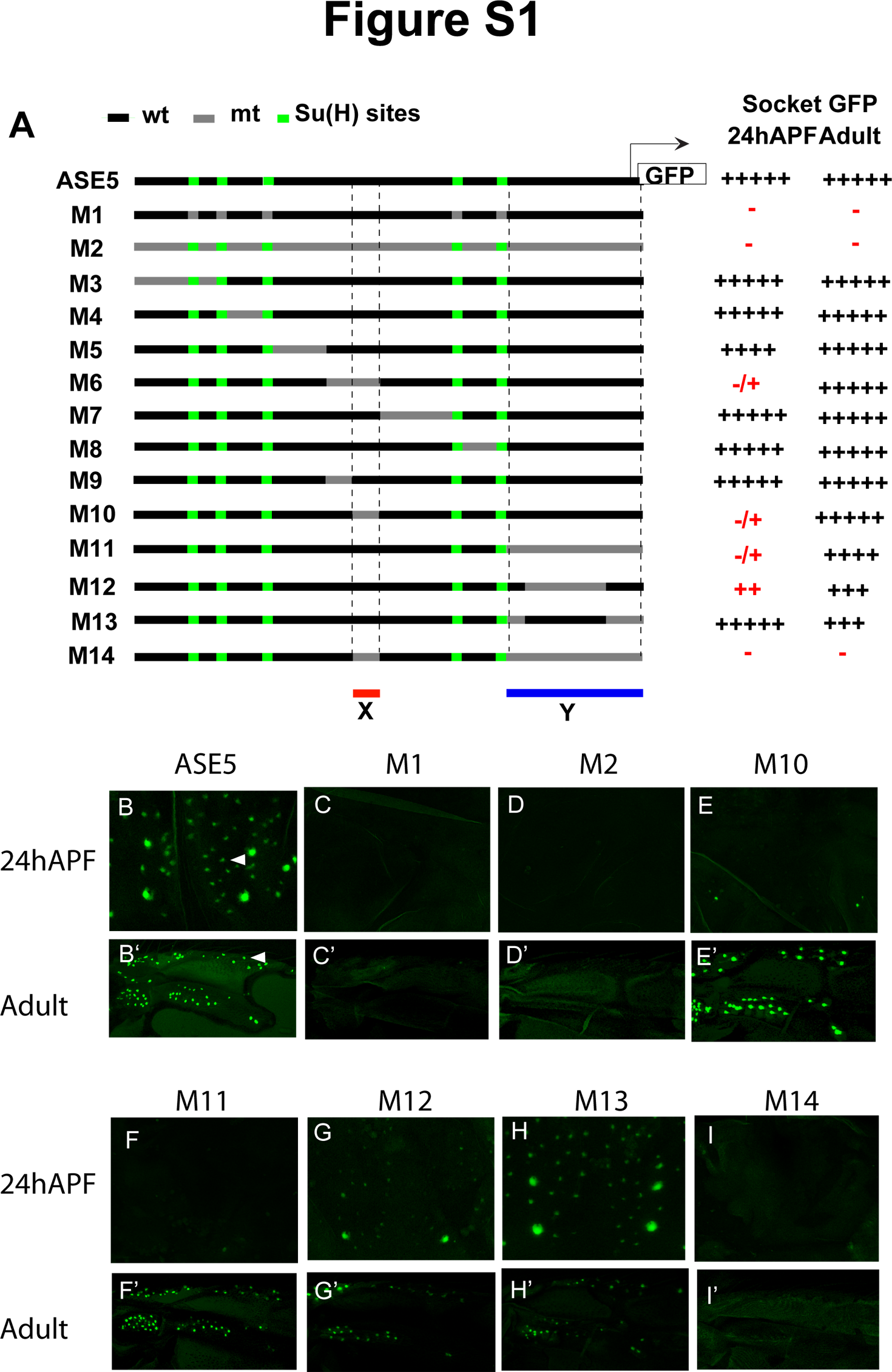

Supplement: Figure S1 — Identification of functionally important sequence elements in ASE5. (A) Diagrams of scanning mutagenesis variants of ASE5 tested in GFP reporter gene constructs. The module's five Su(H) binding sites are marked in green; other wild-type (wt) sequences are shown in black, while mutant (mt) sequence (see Materials and Methods) is marked in gray. All constructs are of the same size as wild-type ASE5. Observed levels of GFP expression in socket cells are summarized at right. Wild-type ASE5 is scored as very strong (+++++); other constructs vary from very strong to moderate (+++) to very weak (+). Constructs that fail to drive detectable GFP expression are indicated as negative (−). (B–I, B′–I′) Effects of scanning mutagenesis on the activity of ASE5 are examined in nascent socket cells of notum microchaetes at 24 hours APF (B–I; see arrowhead in B), and in mature socket cells in the anterior proximal wing in adults (B′–I′; see arrowhead in B′); results are summarized in A. Mutating all five Su(H) binding sites (M1) completely abolishes the activity of ASE5 (C, C′). Likewise, mutating all sequences between the Su(H) sites (M2) abolishes ASE5's activity (D, D′). Separately mutating two distinct fragments [fragment X or Y (see A); M10 and M11, respectively] results in severe reduction of ASE5's activity in nascent socket cells, but not in adult socket cells (E–F, E′–F′). Mutating both fragments X and Y (M14) results in complete loss of ASE5-GFP expression at both stages (I, I′). (TIF) [file pgen.1002796.s001.tif]

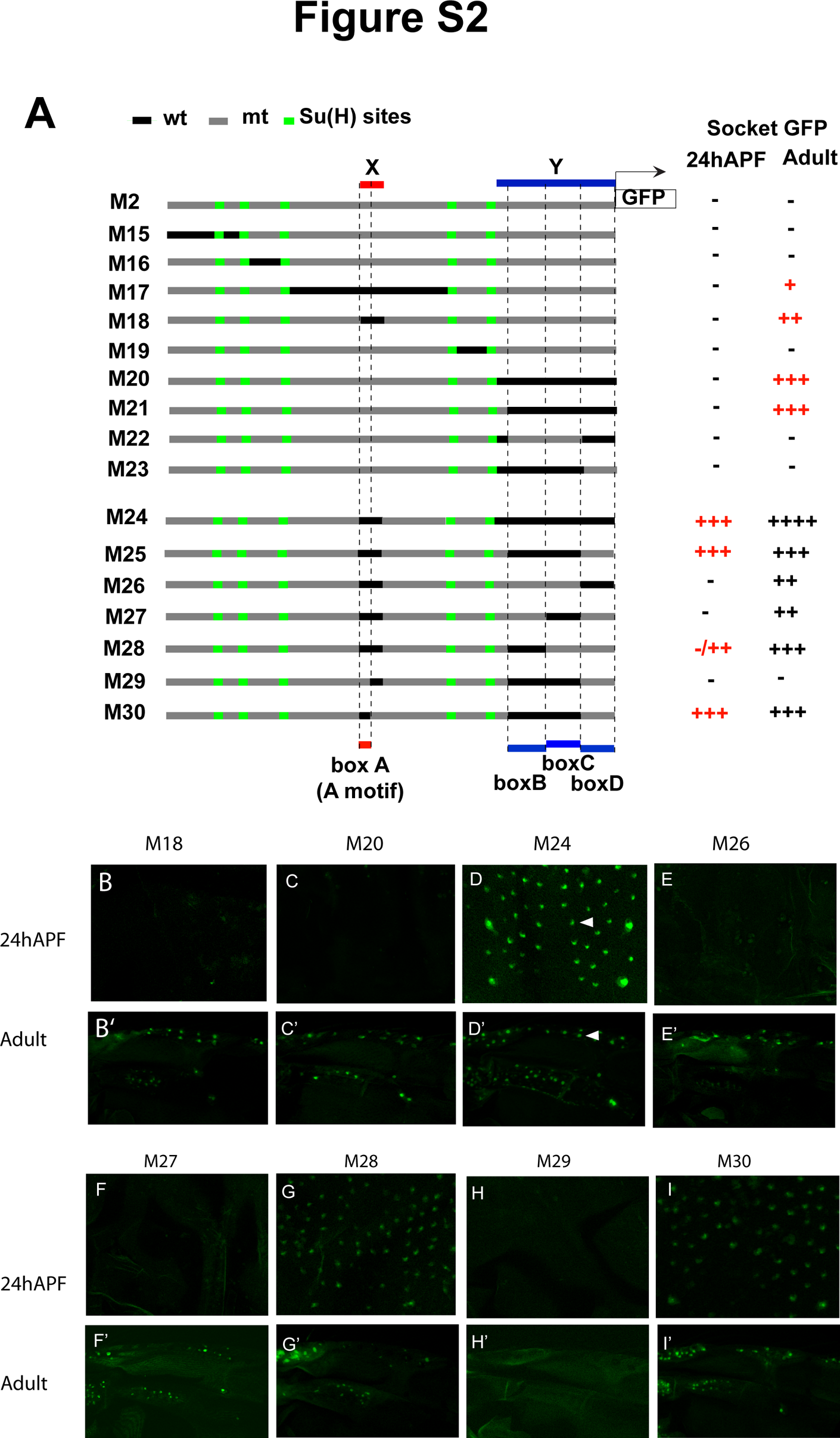

Supplement: Figure S2 — Combinatorial activation of ASE5 in the developing socket cell. (A) Diagrams of ASE5 mutants containing wild-type sequences of the five Su(H) binding sites (marked in green), along with wild-type (wt) sequences of other specific segments of the enhancer (shown in black); all other sequences are mutant (mt, marked in gray). All variants are of the same size as wild-type ASE5. Enhancer activities of each fragment were tested in GFP reporter gene constructs. Observed levels of GFP expression in socket cells are summarized at right, using the same semi-quantitative scoring system as in Figure S1. (B–I, B′–I′) Reporter gene expression was examined in nascent socket cells of notum microchaetes at 24 hours APF (B–I; see arrowhead in D), and in mature socket cells in the anterior proximal wing in adults (B′–I′; see arrowhead in D′); results are summarized in (A). Inputs from the Su(H) sites plus either fragment X or Y (M18, M20) are sufficient to activate GFP expression in adult socket cells, but are insufficient in nascent socket cells (B–C, B′–C′). Inputs from the Su(H) sites plus both X and Y (M24) are sufficient to activate GFP expression in socket cells at both stages (D, D′). Three sub-elements of Fragment Y (boxes B, C, and D) each contribute to its activity [B: compare M18 (B, B′) and M28 (G, G′); C: compare M25 (see A) and M28 (G, G′); D: compare M20 (C, C′) and M23 (see A)]. Fragment X's function maps to an 11-bp element called the A motif [box A; compare M25 (see A), M29 (H, H′), and M30 (I, I′)]. Fragments X and Y were each used as bait in yeast one-hybrid screens (see Text S1, Table S1, and Table S2). (TIF) [file pgen.1002796.s002.tif]

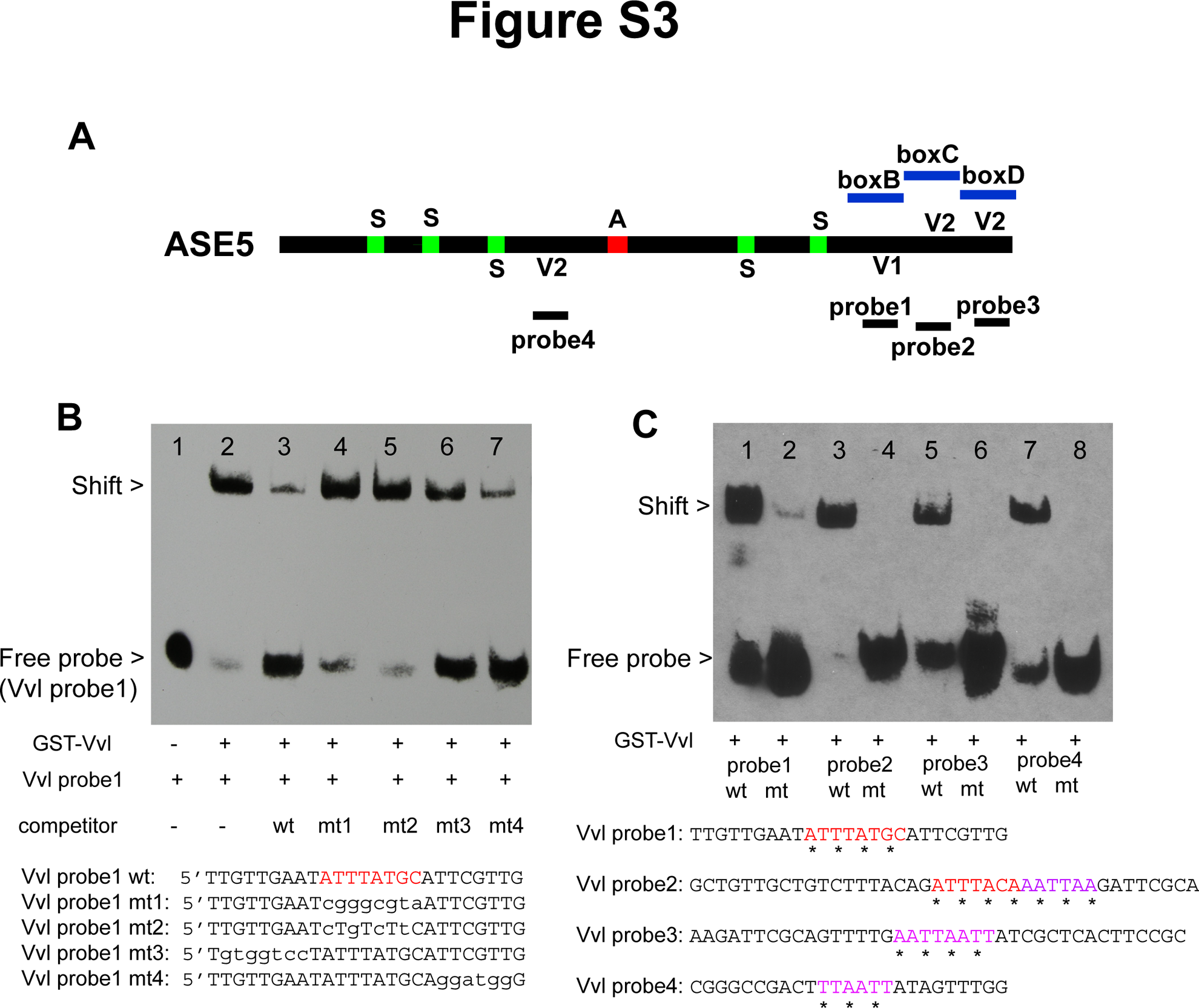

Supplement: Figure S3 — ASE5 contains multiple Vvl binding sites. (A) Diagram of ASE5, showing positions of strong Vvl binding sites. Su(H) binding sites (S) are marked in green; the A motif is shown in red; boxes B–D are shown in blue. The single type 1 Vvl octamer site, which conforms to the definition RYRYAAAT, is located within box B and is indicated as V1. The type 2 Vvl sites, which all contain the hexamer AATTAA, are indicated as V2; note that one such site is located within both box C and box D. Positions of oligonucleotide sequences used as probes in electrophoretic mobility shift assays (EMSAs) are shown. (B) Competition EMSA using Vvl probe 1 and purified GST-Vvl. A 500-fold excess of unlabeled oligonucleotides were used as competitors (wt, wild-type; mt, mutant). Positions of free and bound probe are indicated. In the probe sequences below, wild-type bases are in upper case; mutant positions are in lower case; the wild-type V1 motif is shown in red. (C) Direct-binding EMSA using wild-type and mutant Vvl probes 1–4 and GST-Vvl. Type 1 Vvl sites are shown in red; type 2 sites are in purple. Mutated bases in mutant probes are indicated with asterisks. Note that probe 2 includes a strong type 2 Vvl binding site and an overlapping weak type 1 site that is a one-base mismatch to the RYRYAAAT motif definition. Both sites are mutated in probe2mt. (TIF) [file pgen.1002796.s003.tif]

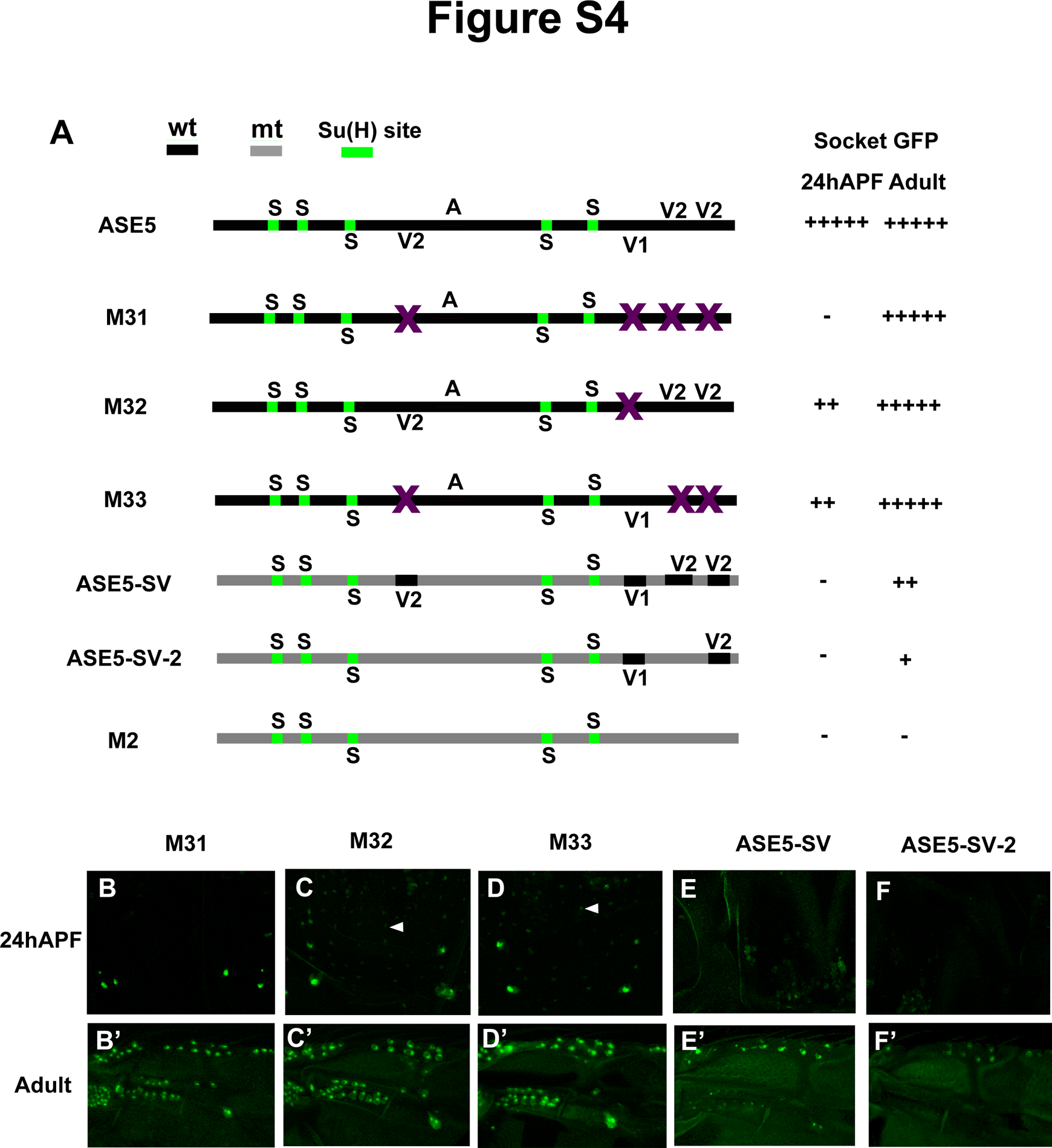

Supplement: Figure S4 — Necessity and sufficiency of Vvl binding sites for ASE5's function. (A) Diagram showing wild-type ASE5, three variants (M31–M33) bearing mutated Vvl sites, and two variants (ASE5-SV and ASE5-SV-2) testing the sufficiency of Vvl sites to synergize with Su(H) sites. ASE5M2, which retains in wild-type form only the five Su(H) sites, is also shown (see Figure S1). Su(H) binding sites (S) are marked in green; the position of the A motif (A) is also shown. Type 1 and type 2 Vvl binding sites are indicated as V1 and V2, respectively. Wild-type (wt) segments of the enhancer are shown in black; mutant (mt) segments are marked in gray. All variants are of the same size as wild-type ASE5. Enhancer activities of each variant were tested in GFP reporter gene constructs; observed levels of GFP expression in socket cells are summarized at right, using the same semi-quantitative scoring system as in Figure S1. (B–F, B′–F′) Reporter gene expression was examined in nascent socket cells of notum microchaetes at 24 hours APF (B–F; see arrowheads in C, D), and in mature socket cells in the anterior proximal wing in adults (B′–F′); results are summarized in (A). (B, B′) Mutating the four Vvl motifs shown in A (M31; purple X's) results in loss of ASE5 activity in nascent, but not adult, socket cells. (C–D, C′–D′) Mutating only the type 1 or type 2 Vvl binding sites (M32, M33) greatly weakens, but does not eliminate, ASE5's activity in nascent socket cells; activity in adult socket cells is unaffected. (E–F, E′–F′) Retaining the wild-type sequences of only the five Su(H) sites and either all four (ASE5-SV; E, E′) or just two (ASE5-SV-2; F, F′) Vvl sites is sufficient to drive GFP expression in adult socket cells. (TIF) [file pgen.1002796.s004.tif]

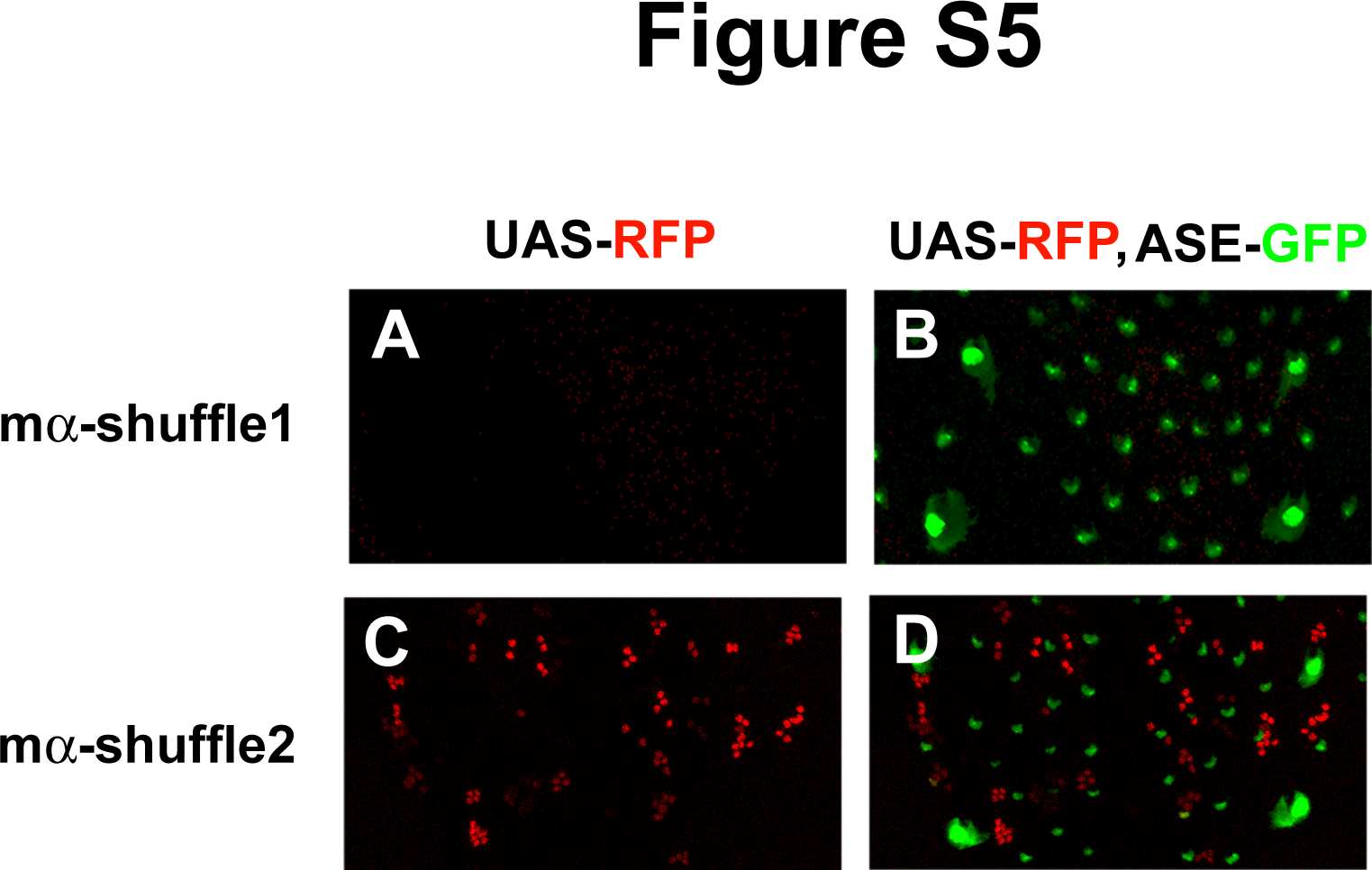

Supplement: Figure S5 — mα-shuffle1 and mα-shuffle2 are not active in nascent socket cells. (A–D) RFP expression (red) driven (using the GAL4-UAS system) by two mα enhancer variants, mα-shuffle1 and mα-shuffle2 (see Figure 5), in the pupal notum at 26 hours APF. In merged images (B, D), socket cells are identified by expression of an ASE-GFP reporter gene (green) [10]. As in the adult (see Figure 5), mα-shuffle2, but not mα-shuffle1, drives limited RFP expression in surrounding epidermal (probably non-SOP) cells. Neither variant drives expression in nascent socket cells. (TIF) [file pgen.1002796.s005.tif]

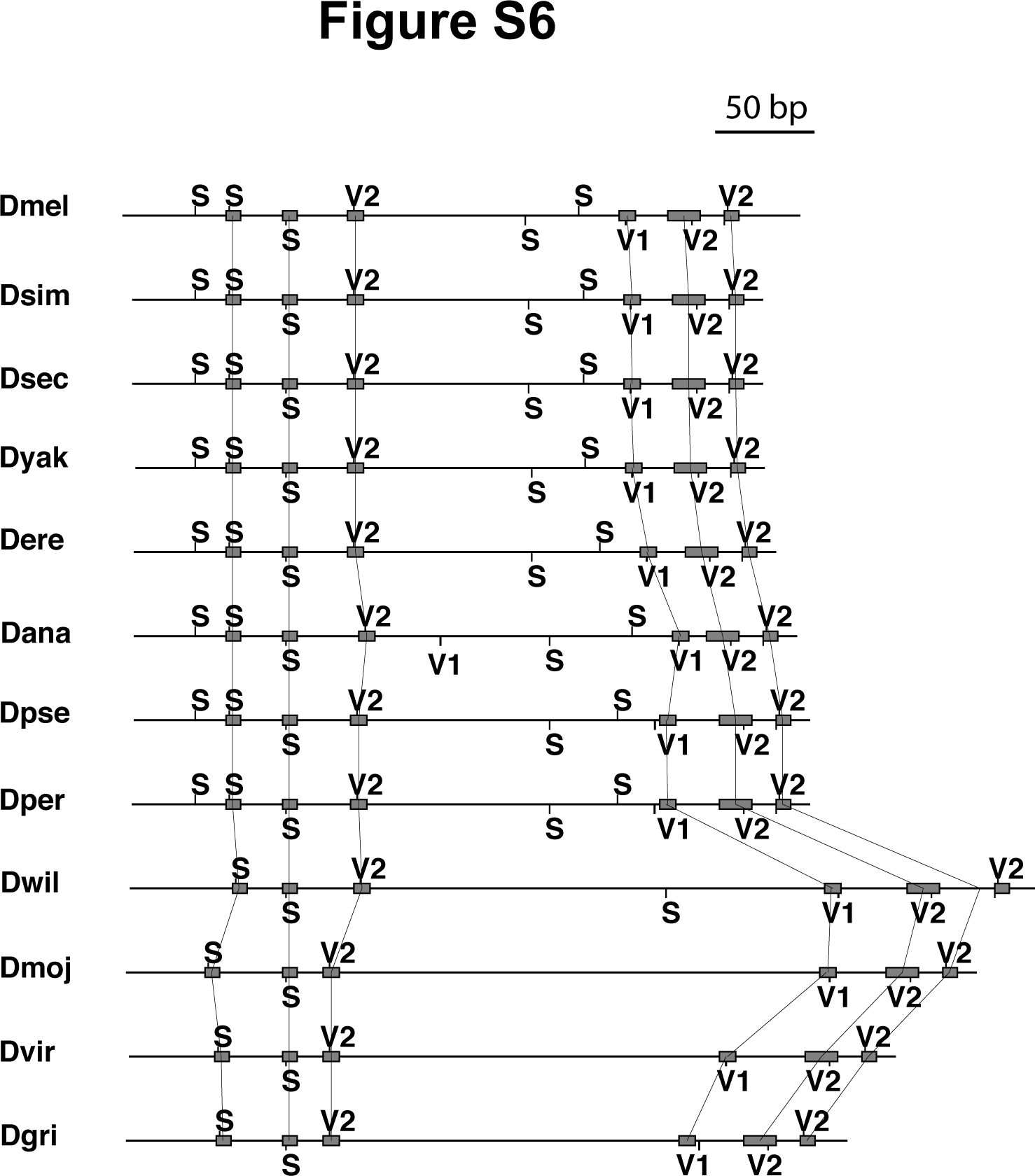

Supplement: Figure S6 — Alignment of ASE5 enhancer sequences from 12 Drosophila species. ASE5 enhancer region sequences were retrieved from the UCSC genome browser (genome.ucsc.edu) and aligned using GenePalette (www.genepalette.org) [28]. Filled boxes represent perfectly conserved words of 9 bases or longer. S: Su(H) binding sites (YGTGDGAA, TGTGTGAA omitted). V1: Type 1 Vvl binding site (RYRYAAAT). V2: Type 2 Vvl binding site (AATTAA). Note the complete conservation of the four Vvl sites (one V1 and three V2) identified in Dmel. (TIF) [file pgen.1002796.s006.tif]

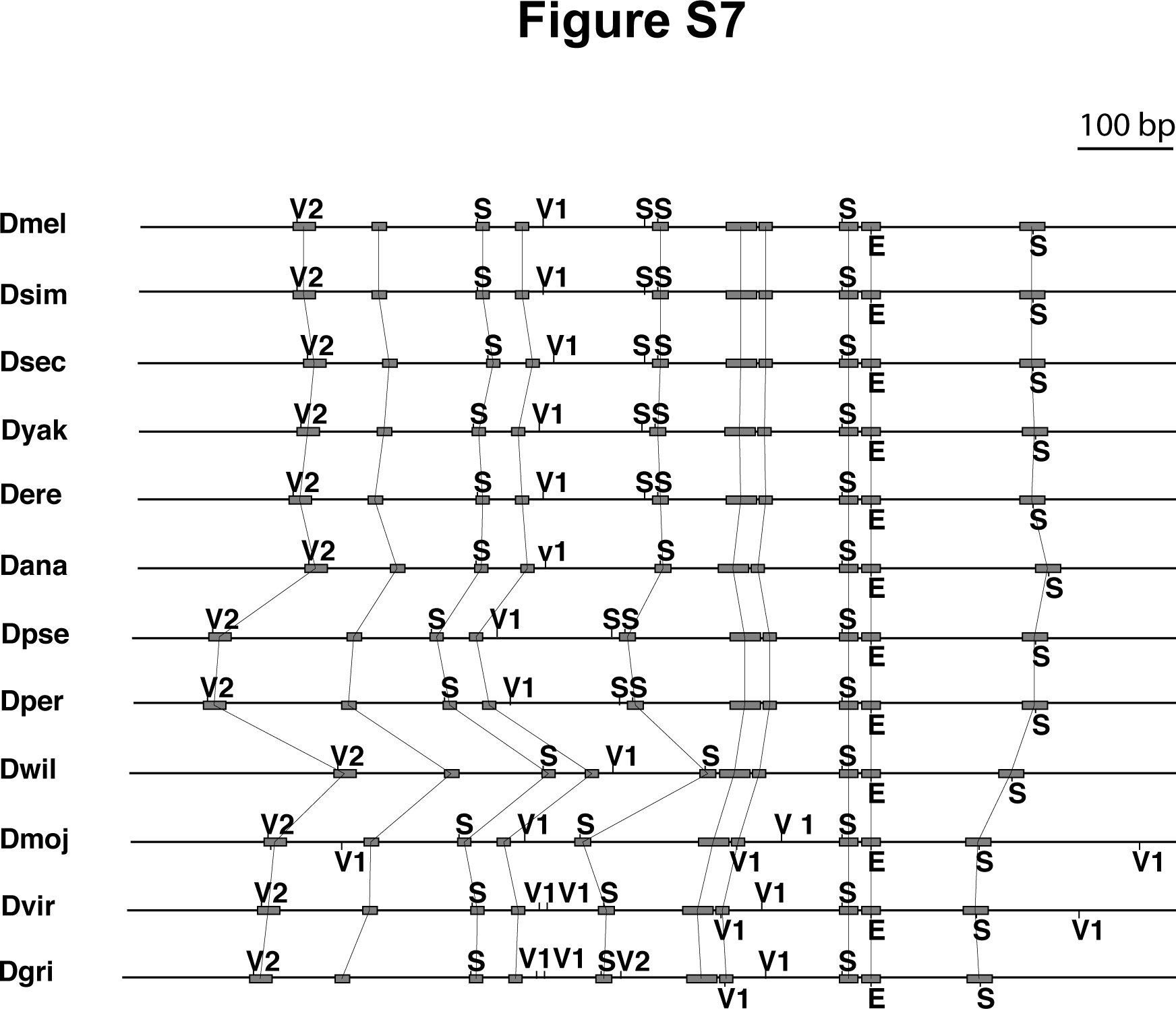

Supplement: Figure S7 — Alignment of mα enhancer sequences from 12 Drosophila species. mα enhancer region sequences were retrieved from the UCSC genome browser (genome.ucsc.edu) and aligned using GenePalette (www.genepalette.org) [28]. Filled boxes represent perfectly conserved words of 10 bases or longer. S: Su(H) binding sites (YGTGDGAA, TGTGTGAA omitted). V1: Type 1 Vvl binding site (RYRYAAAT). V2: Type 2 Vvl binding site (AATTAA). As previously described [11], the enhancer's Su(H) sites are conserved, with the exception of the second site from the left (S4 [11]) which is lost in Dana, Dwil, Dmoj, Dvir, and Dgri. Note the strict conservation of sequence and spacing in the module's S+E motif combination [11]; this appears to be a critical “grammar” element within the enhancer (this study). Also note that, with the exception of V1 in Dana [which has a single-base mismatch to the definition (ATACAAAC), shown as lower-case “v”], both Vvl binding sites identified in Dmel are fully conserved. (TIF) [file pgen.1002796.s007.tif]
