# Supplementary material for: Role of Architecture in the Function and Specificity of Two Notch-Regulated Transcriptional Enhancer Modules
Source: PLoS Genet. 2012 Jul 5;8(7):e1002796. doi: 10.1371/journal.pgen.1002796 (PMC3390367; doi:10.1371/journal.pgen.1002796)
Supplement: Table S1 — Yeast one-hybrid screen using Fragment Y as bait. (PDF) [file pgen.1002796.s008.pdf]

Table S1. Yeast one-hybrid screen using Fragment Y as bait.

Gene	Number of independent clones
vvl	13
yan/aop	1
D	1
zfh2	1
CG43444	1
CG8372	1
tyf	1
CG14971	1
Cenp-C	1
eIF-4a	1
TfIIFα	1
14-3-3ε	1
Ost48	1
Nrg	1
mim	1
Ribosomal RNA/proteins	44
Other (matched to reverse strand)	4
