# Supplementary material for: Role of Architecture in the Function and Specificity of Two Notch-Regulated Transcriptional Enhancer Modules
Source: PLoS Genet. 2012 Jul 5;8(7):e1002796. doi: 10.1371/journal.pgen.1002796 (PMC3390367; doi:10.1371/journal.pgen.1002796)
Supplement: Table S2 — Yeast one-hybrid screen using Fragment X as bait. (PDF) [file pgen.1002796.s009.pdf]

Table S2. Yeast one-hybrid screen using Fragment X as bait.

Gene	Number of independent clones
tyn	2
bnb	1
cg	1
hth	1
tap	1
dome	1
akirin	1
plexB	1
CG40191	1
CG2519	1
lig	1
WDR79	1
AGO1	1
EF1α48D	1
Sulf1	1
Akap200	1
yu	1
βTub60D	1
Fit1	1
nAcRβ-64B	1
CD98hc	1
CG5080	1
endos	1
Ribosomal RNA/proteins	50
Other (matched to reverse strand)	15
