# Supplementary material for: Role of Architecture in the Function and Specificity of Two Notch-Regulated Transcriptional Enhancer Modules
Source: PLoS Genet. 2012 Jul 5;8(7):e1002796. doi: 10.1371/journal.pgen.1002796 (PMC3390367; doi:10.1371/journal.pgen.1002796)
Supplement: Text S1 — Supplemental Methods. (PDF) [file pgen.1002796.s010.pdf]

Supporting Information for:

Role of Architecture in the Function and Specificity of Two Notch-Regulated Transcriptional Enhancer Modules

Feng Liu and James W. Posakony
Division of Biological Sciences/CDB, University of California San Diego, La Jolla, California, United States of America

Text S1
Supplemental Methods

Sequence of ASE5

ASE5 was originally defined by a 372-bp Bsu36I-AseI genomic DNA fragment downstream of the 3' UTR of *Su(H)* [10]. In the present study, we included an additional 19-bp sequence at the 3' end (underlined) because this segment is part of a highly conserved sequence block. Later we found that adding this 19-bp sequence also allowed us to include the entirety of a Vvl type 2 binding site (AATTAA). Mutant variants of ASE5 were created by introducing non-complementary transversions (A <-> C and G <-> T) into target regions. As an example, the sequence of ASE5M2, in which all bases between the five Su(H) binding sites were mutated, is shown below. The sequences of other ASE5 variants are available on request.

In the sequences below, Su(H) binding sites are shaded in green, box A in red, and Vvl binding sites in yellow. Mutated bases in ASE5M2 are shown in lower case; the proneural protein binding site introduced by the mutagenesis is shown in bold. The Bsu36I and AseI restriction sites are shown in italics.

>Dmel_ASE5

*CCTAAGG*TGGAGCGCTGGGGAGGATTGGTTGCCTTAAATTGCTGTGGGAATATTACCCAGTATC
GTGTGAATATTTTTAAACAAAACAAGTGCAGCTCTTCCCACGATGGTTTGCCTCAACCCCCTCG
GACGGGCCGACTTTAATTATAGTTTGGTCTGGAATTGCTGAACGCGAAGCTAAGAAGCAAATAT

ACATAGATATATAACACTATTGTTTCCGTTTTGGCCAAGTTAAGGAGCAGAATATTCTCACAGC
CCAAAAAGCCGCTGCCGAGCTGCTGTGAGAATTTTTACTGCCTTTGTTGAATATTTATGCATTT
GTTGCTGTTGCTGTCTTTACAGATTTACAAATTAAGATTCGCAGTTTTG*AATTAAT*TATCGCTC
ACTTCCG

>Dmel_ASE5M2
*CCTAAGG*gttctatagtttcttcggttggtaaggcccggtCTGTGGGAATcggcaaactgcTC
GTGTGAATcgggggcccaccc**cacctgt**actagCTTCCCACGAgttgggtaagaccaaaaagat
tcatttaatcagggccggcgctgggttgagttccggtagtccatatcctggcctcctacccgcg
cacgctcgcgcgccacagcggtgggaatggggttaacctggccttctactccgATTCTCACAGa
aacccccctaatagtaatctagtCTGTGAGAATggggcagtaagggtggtccgcgggcgtacggg
tggtagtggtagtgagggcactcgggcacccggcctcggatactggggtccggccggcgataga
caggaat

Design of primer sequences for synthesizing ASE5

To accelerate the introduction of large blocks of mutations (non-complementary transversions: A <-> C and G <-> T) into ASE5, we synthesized the enhancer fragment de novo by recursive PCR [22], using nine forward primers and nine reverse primers as listed below. The forward primer sequences do not overlap with each other, but each primer overlaps with two adjacent reverse primers by 20 bases each. The 5' and 3' end primers include EcoRI and BamHI restriction sites, respectively. To generate ASE5 mutants or ASE5-shuffle variants, primers containing mutated or shuffled sequences were used instead of their wild-type counterparts in the recursive PCR.

The primers used for synthesizing wild-type ASE5 are shown below; primer sequences used for making ASE5 mutants and other variants are available upon request. Lower-case letters denote restriction sites (bold) and end-protecting nucleotides.

Forward primers for synthesizing ASE5:

ASE5-F1(EcoRI): ccg**gaattc**CCTAAGGTGGAGCGCTGGGGAGGATTGGTTGCCTTA

ASE5-F2: AATTGCTGTGGGAATATTACCCAGTATCGTGTGAATATTTTTAA

ASE5-F3: ACAAAACAAGTGCAGCTCTTCCCACGATGGTTTGCCTCAACCCC

ASE5-F4: CTCGGACGGGCCGACTTTAATTATAGTTTGGTCTGGAATTGCTG

ASE5-F5: AACGCGAAGCTAAGAAGCAAATATACATAGATATATAACACTAT

ASE5-F6: TGTTTCCGTTTTGGCCAAGTTAAGGAGCAGAATATTCTCACAGC

ASE5-F7: CCAAAAAGCCGCTGCCGAGCTGCTGTGAGAATTTTTACTGCCTT

ASE5-F8: TGTTGAATATTTATGCATTTGTTGCTGTTGCTGTCTTTACAGA

ASE5-F9: TTTACAAATTAAGATTCGCAGTTTTGAATTAATTATCGCTCACTT

Reverse primers for synthesizing ASE5:

ASE5-R1: GTAATATTCCCACAGCAATTTAAGGCAACCAATCCTCCCC

ASE5-R2: GTAATATTCCCACAGCAATTTAAGGCAACCAATCCTCCCC

ASE5-R3: TTAAAGTCGGCCCGTCCGAGGGGGTTGAGGCAAACCATCG

ASE5-R4: TTGCTTCTTAGCTTCGCGTTCAGCAATTCCAGACCAAACT

ASE5-R5: ACTTGGCCAAAACGGAAACAATAGTGTTATATATCTATGT

ASE5-R6: GCTCGGCAGCGGCTTTTTGGGCTGTGAGAATATTCTGCTC

ASE5-R7: AAATGCATAAATATTCAACAAAGGCAGTAAAAATTCTCAC

ASE5-R8: TGCGAATCTTAATTTGTAAATCTGTAAAGACAGCAACAGC

ASE5-R9 (BamHI): cgc**ggatcc**CGGAAGTGAGCGATAATTAATTC

Yeast one-hybrid screens

We performed two separate yeast one-hybrid screens, with either Fragment X or Fragment Y of ASE5 (see Figure S1A) as bait, using the Matchmaker Yeast One-Hybrid System (Clontech). The cDNA library used in the screen was prepared from stage-13 embryos, when embryo socket cells are newly born.

The Fragment Y bait was generated by PCR; the Fragment X bait by annealing two synthetic complementary oligonucleotides (Integrated DNA Technologies). A cDNA library was generated from 2 μg of total RNA from stage-13 embryos, and was cloned

into the prey plasmid as a fusion with GAL4AD coding sequences by in vivo recombination according to the manufacturer's instructions. High-efficiency yeast transformation was performed as described [29]. Positive clones were selected on synthetic defined media -Trp -Leu -His, plus 10 mM 3-AT. In total, 1.2 million clones were screened with bait Y, and 0.6 million clones with bait X. For each screen, 100 independent clones from selection plates were serially streaked three times on fresh selection plates, after which the cDNA insert was amplified from individual clones by PCR and sequenced. Identity of the clones was determined by BLAST analysis against the *Drosophila melanogaster* genome.

With Fragment Y as bait, a significant fraction of positively selected clones (13/75) contained a cDNA fragment encoding Ventral veins lacking (Vvl), a POU-HD transcription factor [30,31]. By contrast, the screen using Fragment X as bait did not yield obvious candidates. See Table S1 and Table S2 for details.

Identification of Vvl binding motifs in ASE5

We found that an eight-nucleotide motif (ATGCAAAT) located in ASE5 Fragment Y perfectly matches a previously characterized Vvl binding site [12]. Using an electrophoretic mobility shift assay (EMSA), we confirmed that this motif, designated V1, is bound specifically in vitro by a purified GST-Vvl fusion protein (see below and Figure S3). To determine if other Vvl binding sites are contained within ASE5, we used double-stranded oligonucleotides covering the entire ASE5 as competitors in the EMSA, which led to the discovery that the AATTAA motif is also bound strongly by GST-Vvl (see Figure S3).

Electrophoretic mobility shift assays (EMSAs)

The complete *vvl* coding sequence was obtained by PCR from a cDNA library of stage-13 embryos (strain w^{1118}), and cloned into vector pGEX-5X-2 (GE Healthcare Life Sciences). The GST-Vvl fusion protein was expressed in *E. coli* strain BL21, and purified according to the manufacturer's instructions. The concentration of the purified protein was estimated by comparing it with BSA standards in a Coomassie-stained 4-20% SDS-PAGE gel (Bio-Rad Laboratories, Inc.).

Oligonucleotides used for EMSA probes were labeled with Biotin-11-UTP and annealed in vitro according to the manufacturer's instructions (Thermo Scientific). For each EMSA reaction, approximately 10 μg of purified GST-Vvl was incubated with 20 pmol of labeled probe for 20 minutes at room temperature. Free and bound probes were separated on 5% non-denaturing polyacrylamide gels (Bio-Rad) and detected using the LightShift Chemiluminescent EMSA kit according to the manufacturer's instructions (Thermo Scientific). For competition assays, 10 nmol of unlabeled oligonucleotides (500-fold excess) were included in each reaction mix.

Sequences of synthetic enhancers based on ASE5

The synthetic enhancer ASE5-shrink is composed of the essential sequences of ASE5: the five Su(H) binding sites, box A, and box B. In the sequences below, Su(H) motifs are shaded in green, box A in red, and Vvl binding sites in yellow. The other three synthetic enhancers (ASE5-shrinkAm, ASE5-shrinkBm, and ASE5-shrinkABm) contain mutations that disrupt the function of either or both box A and the Vvl motif in box B; in the sequences below, mutated bases are shown in lower case.

```
>ASE5-shrink
ATTGCTGTGGGAATATTAAGTATCGTGTGAATATTTAGCTCTTCCCACGATGGTAACGCGAAGC
TGAATATTCTCACAGCGCTGCTGTGAGAATTTTTTGTTGAATATTTATGCATTTGTTGCTGTT
```

```
>ASE5-shrinkAm
ATTGCTGTGGGAATATTAAGTATCGTGTGAATATTTAGCTCTTCCCACGATGGTccCtaGActC
TGAATATTCTCACAGCGCTGCTGTGAGAATTTTTTGTTGAATATTTATGCATTTGTTGCTGTT

>ASE5-shrinkBm
ATTGCTGTGGGAATATTAAGTATCGTGTGAATATTTAGCTCTTCCCACGATGGTAACGCGAAGC
TGAATATTCTCACAGCGCTGCTGTGAGAATTTTTTGTTGAATcTgTcTtCATTTGTTGCTGTT

>ASE5-shrinkABm
ATTGCTGTGGGAATATTAAGTATCGTGTGAATATTTAGCTCTTCCCACGATGGTccCtaGActC
TGAATATTCTCACAGCGCTGCTGTGAGAATTTTTTGTTGAATcTgTcTtCATTTGTTGCTGTT
```

Sequences of the mα enhancer and variants

The mα enhancer was described previously [11]; its sequence is shown below. Su(H) motifs are shaded in green, Vvl binding sites in yellow, and the E-box in blue. The enhancer fragment was PCR-amplified using the following primers:

Forward (EcoRI): ccg**gaattc**CCCTAGCCAAACAACAAGTCATTAAAACGT

Reverse (BamHI): cgc**ggatcc**CGACAGAGAGGGCGAGAACGGACCCCTGCC

To create the mα-shuffle1-3 variants, two segments centered on each indicated binding site (underlined) were switched in position: Proneural protein binding site, 5'-AGGAA<u>CACCTGC</u>CCCGT; most proximal Su(H) site, 5'-ATTGT<u>TTCCCACA</u>CTCGT; V1 site, 5'-TGCCT<u>ATACAAAT</u>AGAAG; V2 site, 5'-GGG<u>AATTAATTAA</u>AAT.

The mαA variant was created by introducing three point mutations into an 11-bp sequence in the mα enhancer (AACCCCAAGAT, shown in bold in the sequence below), thus converting it to the sequence of the A motif (AACGCGAATCT).

The segments underlined in the mα enhancer sequence below were joined to make the mα-shrink enhancer; the "E box" segment was omitted in the construction of the mα-shrinkΔE and mα-shrinkΔE-Vm variants. Both the mα-Vm and mα-shrinkΔE-Vm

variants include the same point mutations in the Vvl binding motifs; these are shown in lower case in the mα-shrinkΔE-Vm sequence below.

>Dmel_mα_enhancer
CCCTAGCCAAACAACAAGTCATTAAAACGTATCTCTAAGTACGCGTTCGTATCTCGCCCTCAGC
TCGCTTTGCACACACTTTCTCCCTGCGCCTTATCGGGTAATCCCTCTCCCTTGAAACAATATTG
AAGAAACCCTCCCTATCGCTCCACACGTTCAGCACACTTGGGCCGCAAGAAAGGGAATTAATTA
AAATATCTATAAGATTCCCAGCTCACCCCTGGGGAGTGTTTCCAAATTGAAGACAAGTGGCAAG
TCCGTATTTTATTGGTGGTGGTTTCGATGGTGCTAGTGAATAGTGGTAAATGGATTCATCGAGC
CCTGTGGGAAAGTTGGAAATCAAAACACCATAACAAGTGATTCGAGATGCCTATACAAATAGAA
GATCC**AACCCCAAGAT**CCCTTATGCCCTTTCATATGCACGAAACCAGAGCCAGGACGAAGCAAT
GTGTGGGAATGCGTGGGAATGGTCCTGGGGATTCGAAACTCAGAAACGGTCCCCTATCCCTGCT
TTTATGTTGATTGCCCATTAGGAATACAATTTGCAACTCCTTTTCCATGATGTGATCCCCTTGT
TGATCCTGGGCTTCCTTCGGTGTCGTGAGAAATTTTACCAAGGAACACCTGCCCCGTATCGGTG
TGTCCTGGATTGCCAGTGTGTTGGTGATTTTTTTCATGTCCTCTGGGGATTTCACGCAGCACTT
GAATACGTTTCCGAAAATTTTGAGGCCAGCGAAAAATTGTTTCCCACACTCGTCGGACAGTTGC
AGTTTTTGTAGGGGTTTGTTTCCTTTGCCGATGCACTCTTCTCTCGTCTCATCCCGTCTCGTCT
TTACCTGCTCCTGGAACTTCCCCCGACTCCTGGCGAGCGTCGTATTTCAGGTTCTCCGCCCCGA
TTATCTCGGCTCTTTGAGGGCGAGGGTCTGCGTACTCCGTTTCTGTCTCTGTTTGTATTCCCCT
CCCTGTCTGCGGTGCGTGTCGCTGGCAGGGGTCCGTTCTCGCCCTCTCTGTCG

>mα-shrink
GGGAATTAATTAAAATAGCCCTGTGGGAAAGTTGTGCCTATACAAATAGAAGCAATGTGTGGGA
ATGCGTGGGAATGGTCGGTGTCGTGAGAAATTTTAGGAACACCTGCCCCGTATTGTTTCCCACA
CTCGT

>mα-shrinkΔE
GGGAATTAATTAAAATAGCCCTGTGGGAAAGTTGTGCCTATACAAATAGAAGCAATGTGTGGGA
ATGCGTGGGAATGGTCGGTGTCGTGAGAAATTTTATTGTTTCCCACACTCGT

>mα-shrinkΔE-Vm
GGGAAgTcAgTAAAATAGCCCTGTGGGAAAGTTGTGCCTcTcCcAcTAGAAGCAATGTGTGGGA
ATGCGTGGGAATGGTCGGTGTCGTGAGAAATTTTATTGTTTCCCACACTCGT

Additional References

29. Gietz RD, Schiestl RH (2007) High-efficiency yeast transformation using the LiAc/SS carrier DNA/PEG method. Nat Protoc 2: 31-34.
30. Billin AN, Cockerill KA, Poole SJ (1991) Isolation of a family of *Drosophila* POU domain genes expressed in early development. Mech Dev 34: 75-84.
31. Treacy MN, He X, Rosenfeld MG (1991) I-POU: a POU-domain protein that inhibits neuron-specific gene activation. Nature 350: 577-584.
